# The circadian regulator PER1 promotes cell reprogramming by inhibiting inflammatory signaling from macrophages

**DOI:** 10.1371/journal.pbio.3002419

**Published:** 2023-12-04

**Authors:** Nobuko Katoku-Kikyo, Seunghyun Lim, Ce Yuan, Jinsha Koroth, Yasushi Nakagawa, Elizabeth W. Bradley, Nobuaki Kikyo

**Affiliations:** 1 Stem Cell Institute, University of Minnesota, Minneapolis, Minnesota, United States of America; 2 Department of Genetics, Cell Biology, and Development, University of Minnesota, Minneapolis, Minnesota, United States of America; 3 Bioinformatics and Computational Biology Graduate Program, University of Minnesota, Minneapolis, Minnesota, United States of America; 4 Department of Orthopedic Surgery, University of Minnesota, Minneapolis, Minnesota, United States of America; 5 Department of Neuroscience, University of Minnesota, Minneapolis, Minnesota, United States of America; UNITED KINGDOM

## Abstract

Circadian regulation of gene expression is prevalent and plays critical roles in cell differentiation. However, its roles in the reprogramming of differentiated cells remain largely unknown. Here, we found that one of the master circadian regulators PER1 promoted virus-mediated reprogramming of mouse embryonic fibroblasts (MEFs) to induced neurons (iNs) and induced pluripotent stem cells (iPSCs). Unexpectedly, PER1 achieved this by repressing inflammatory activation of contaminating macrophages in the MEF culture, rather than by directly modulating the reprogrammability of MEFs. More specifically, we found that transduced viruses activated inflammatory genes in macrophages, such as *Tnf* encoding TNFα, one of the central inflammatory regulators and an autocrine activator of macrophages. TNFα inhibited iN reprogramming, whereas a TNFα inhibitor promoted iN reprogramming, connecting the inflammatory responses to iN reprogramming. In addition, macrophages were induced to proliferate and mature by non-macrophage cells serving as feeders, which also supported up-regulation of TNFα in macrophages without virus transduction. Furthermore, the 2 inflammatory responses were repressed by the circadian regulator PER1 in macrophages, making reprogrammability dependent on time-of-day of virus transduction. Similar results were obtained with iPSC reprogramming, suggesting a wide occurrence of macrophage-mediated inhibition of cell reprogramming. This study uncovers mechanistic links between cell reprogramming, bystander inflammatory macrophages, and circadian rhythms, which are particularly relevant to in vivo reprogramming and organoid formation incorporating immune cells.

## Abstract 2

Circadian regulation of gene expression plays critical roles in cell differentiation, but what about reprogramming of differentiated cells? This study shows that contaminating macrophages in fibroblast culture play unsuspected inhibitory roles during reprogramming to neurons and pluripotent stem cells, controlled by the circadian rhythm gene Per1.

## Introduction

Reprogramming of differentiated cells by transcription factors and small molecules has been a major research area in stem cell biology. Since the first report on the reprogramming of mouse embryonic fibroblasts (MEFs) to induced neurons (iNs) by transduction of the 3 neuronal transcription factor genes *Brn2*, *Ascl1*, and *Myt1l* (called BAM hereafter), diverse protocols have been used to reprogram fibroblasts and glia directly to specific subtypes of neurons, such as dopaminergic, GABAergic, and glutamatergic neurons, as well as neuronal precursors in vitro [1–4]. Similar approaches have been applied to in vivo reprogramming of glia to neurons to treat mouse models of brain and spinal cord injuries, neurodegenerative diseases, and retinopathies among others. These studies uncovered reprogramming of signaling pathways, epigenetics, metabolism, and cell cycle in the target cells of reprogramming, contributing to improved reprogramming efficiency and quality of iNs. However, much less attention has been paid to the effects of neighboring non-reprogrammed cells in the same culture dish or tissue. For example, little is known about whether contaminating immune cells play any roles in reprogramming. This missing information is important not only to raise the reprogramming efficiency in vitro but also to understand how reprogramming works in a more physiological and complex environment in vivo. While investigating the roles of circadian regulators in cell reprogramming, we became aware of the inhibitory roles of contaminating macrophages in the reprogramming of MEFs to iNs and to induced pluripotent stem cells (iPSCs). The macrophages’ roles were suppressed by one of the circadian master regulators PER1.

Virtually all mouse tissues examined express intrinsic circadian regulators centered on the CLOCK/BMAL1 transcription factor complex [5,6]. This complex activates thousands of genes, including *Cry1*, *Cry2*, and *Per1-Per3* through binding to the E-box in their promoters. CRY and PER then heterodimerize and directly inhibit the CLOCK/BMAL1 complex, forming the first negative feedback loop. Subsequently, CRY and PER will be degraded by the proteasomal pathway, allowing the CLOCK/BMAL1 complex to resume the circadian cycle. Additionally, CLOCK/BMAL1 activates transcription of the RoR and Rev-Erb genes, which are activators and repressors of the *Baml1* gene, respectively, providing the second feedback loop. Circadian rhythms in various peripheral tissues in a mammalian body are synchronized by the central pacemaker in the suprachiasmatic nucleus in the hypothalamus, which is entrained by the light signal transmitted from the retina. However, ubiquitous peripheral clocks can also be synchronized by other stimulations such as food intake and physical activity.

*Per1*^*-/-*^ mice and *Per2*^*-/-*^ mice are fertile and do not demonstrate major morphological abnormalities but they exhibit a shorter circadian period by 2 h than wild-type (WT) mice and later become arrhythmic in constant darkness [7–10]. A limited sequence identity between PER1 and PER2 (73.4% similarity at the amino acid level) imparts nonredundant functions. Their roles in neuronal development remain largely unknown but several reports imply their relevance to disease pathology. *Per1*^*-/-*^ mice exhibit reduced autophagy and higher neuronal susceptibility to ischemic damages [11,12]. In addition, aged *Per1*^*-/-*^ mice show altered morphology of microglia (macrophages in the central nervous system), up-regulation of presenilin-2, and increased deposition of β amyloid and lipofuscin in the hippocampus, suggesting excessive accumulation of misfolded proteins due to slowed autophagy [13].

Given the prevalence of the circadian feedback loops, it is not surprising that differentiation of many lineages of cells is under circadian regulation [14,15]. For example, we previously showed that CRY2 is important for cell cycle exit and fusion of myoblasts during myotube formation [16]. In addition, PER1 and PER2 regulate myoblast differentiation and muscle regeneration via the IGF2 pathway [17]. In the current study, we hypothesized that cell reprogramming was also controlled by circadian regulators and tested this by applying *Per1*^*-/-*^ and *Per2*^*-/-*^ MEFs to iN and iPSC reprogramming models.

## Results

### *Per1* depletion inhibits iN reprogramming

**Fig 1:**
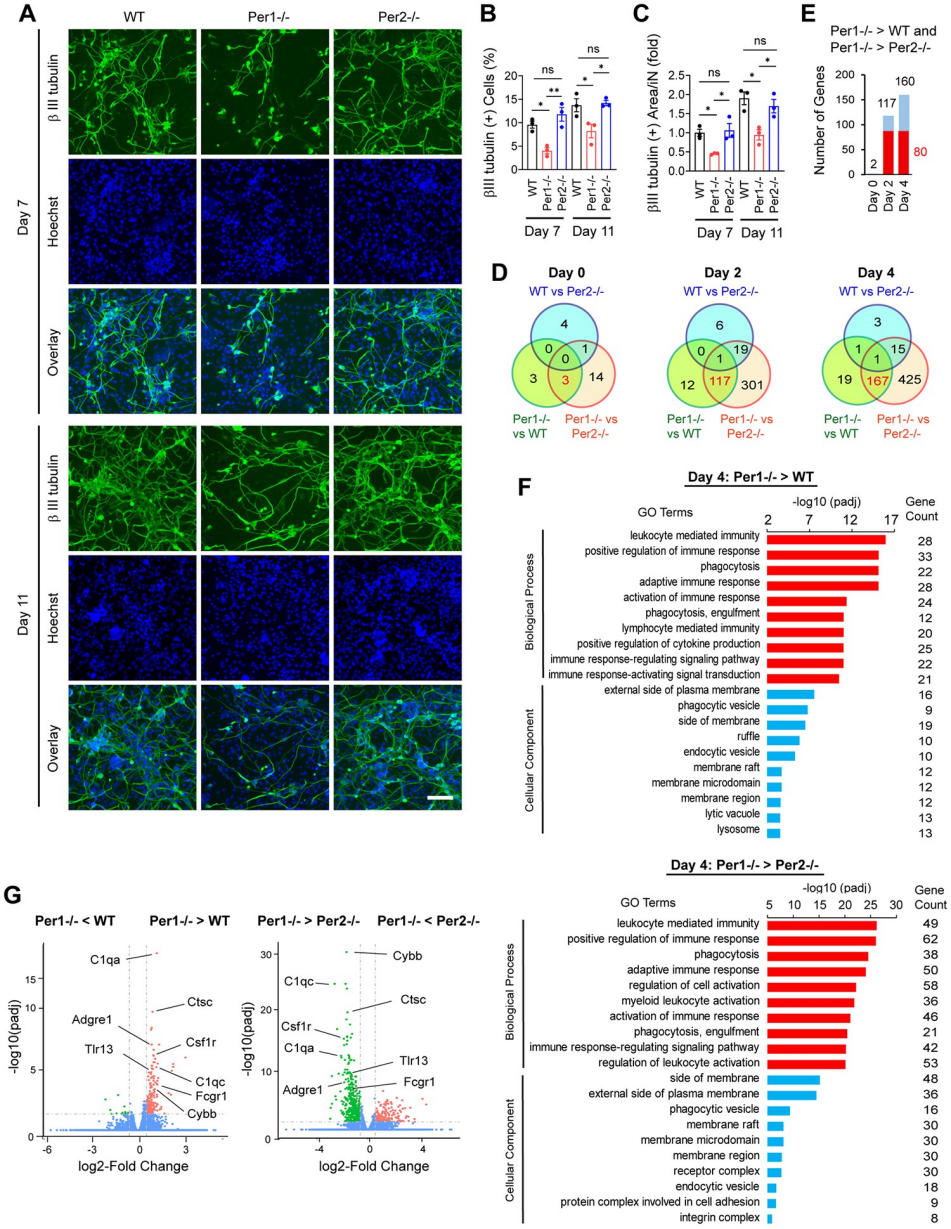
Immunofluorescence staining and transcriptome analyses of iNs prepared from WT, *Per1*^*-/-*^, and *Per2*^*-/-*^ MEFs. (A) Immunofluorescence staining of iNs with βIII tubulin antibody on days 7 and 11. DNA was counterstained with Hoechst 33342. Bar, 100 μm. (B) Percentage of βIII tubulin (+) cells. (C) βIII tubulin (+) neurite area in each βIII tubulin (+) cell. The value with day 7 WT iNs was defined as 1.0. (D) Venn diagrams depicting the numbers of differentially expressed genes (Log2 fold change ≥ 0.58 [≥1.5-fold] and padj ≤0.05) on each day. (E) The numbers of commonly up-regulated genes in *Per1*^*-/-*^ cells in comparison to WT and *Per2*^*-/-*^ cells. Red bars indicate 80 common genes between days 2 and 4. (F) Gene Ontology analysis of the genes up-regulated in *Per1*^*-/-*^ cells in comparison to WT (top) and *Per2*^*-/-*^ cells (bottom) on day 4. (G) Volcano plots for the indicated comparisons on day 4. Macrophage genes with high -log10(padj) values that commonly appeared on the right and left panels are highlighted. * *p* < 0.05 and ** *p* < 0.01 with ordinary one-way ANOVA with Bonferroni’s multiple comparison test; ns indicates statistically not significant. All data are based on biological triplicates. The data underlying this figure can be found in S1 Data. iN, induced neuron; MEF, mouse embryonic fibroblast; WT, wild-type.

To explore the roles of the *Per* genes in cell reprogramming, we compared the efficiency of making iNs from MEFs prepared from WT, *Per1*^*-/-*^, and *Per2*^*-/-*^ mice. We first verified the loss of the PER1 and PER2 proteins in *Per1*^*-/-*^ and *Per2*^*-/-*^ MEFs, respectively, by western blotting (S1A Fig). We then transduced the BAM genes with lentivirus as previously described [2] and assessed successful iN production with immunostaining of βIII tubulin and MAP2. We found that neurites expressing βIII tubulin or MAP2 were less dense in *Per1*^*-/-*^ iNs compared with WT and *Per2*^*-/-*^ iNs despite similar cell densities (Figs 1A and S1B). We quantified this observation in 2 ways. First, the reprogramming efficiency of *Per1*^*-/-*^ iNs defined by the percentage of cells that expressed βIII tubulin or MAP2 was reduced to less than 60% of WT and *Per2*^*-/-*^ iNs (Figs 1B and S1C, red versus others). There was no statistically significant difference in the percentages between WT and *Per2*^*-/-*^ iNs. Second, the areas covered by neurites for each iN were decreased in *Per1*^*-/-*^ iNs compared with WT and *Per2*^*-/-*^ iNs (Figs 1C and S1D). DNA replication was hardly detectable when DNA were labeled with EdU for 6 h; total cell numbers remained unchanged during iN reprogramming across all 3 genotypes (S1E and S1F Fig). Thus, *Per1* depletion decreased the number of iNs as well as neurite development in each iN.

### Bulk RNA-seq indicates activation and maturation of macrophages

We applied days 0, 2, and 4 samples of the 3 genotypes to bulk RNA-seq and obtained the data with the sequence depth of 41 to 61 million reads per sample. This study demonstrated that WT cells were more closely related to *Per2*^*-/-*^ cells than *Per1*^*-/-*^ cells on reprogramming days 2 and 4 (S2A and S2B Fig). Specifically, more than 100 genes were commonly differentially expressed when *Per1*^*-/-*^ cells were compared with either WT cells or *Per2*^*-/-*^ cells each day (Fig 1D). Notably, a vast majority of differentially expressed genes were up-regulated in *Per1*^*-/-*^ cells (117/117 and 160/167 genes on days 2 and 4, respectively) (Fig 1E). The up-regulated genes were enriched with those involved in immune response, phagocytosis, and lysosome (Fig 1F). We focused on 80 genes that were up-regulated on both days (Fig 1E, red), which were reproducibly up-regulated in all biological triplicates (S2C Fig). They were also enriched with genes relevant to innate immunity, phagocytosis, and pro-inflammatory cytokines (TNF and IL-6) (S2D Fig) with a strong representation of macrophage genes (Figs 1G and S2E). They included genes relevant to surface markers for macrophages (*CD11b*, *F4/80*, and *Csfr1* encoding M-CSF receptor), the complement system, and phagocytosis.

### Inflammatory genes are activated in *Per1*^*-/-*^ macrophages

**Fig 2:**
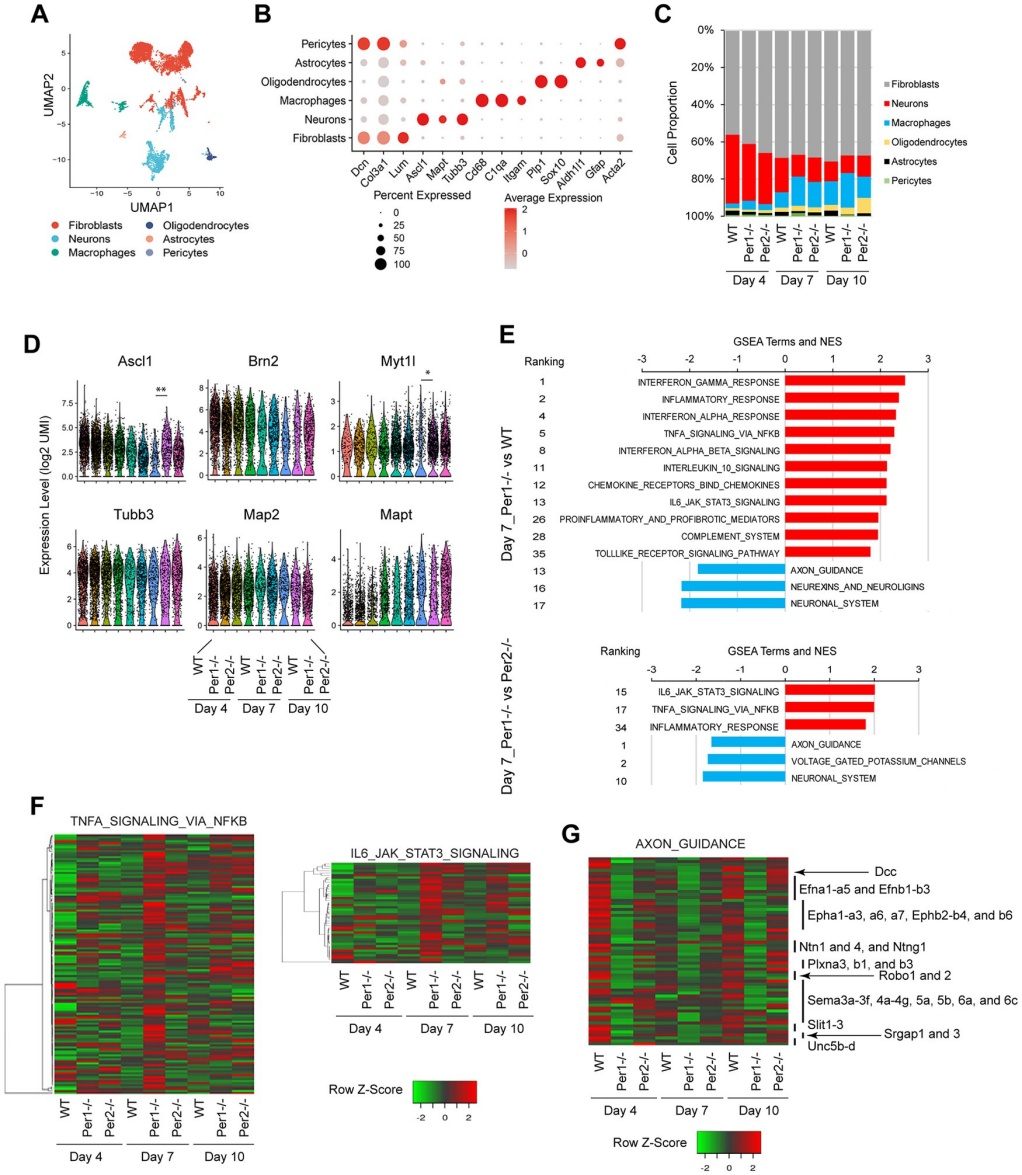
scRNA-seq analyses of iNs and other cells. (A) UMAP combining all 9 samples: WT, *Per1*^*-/-*^, and *Per2*^*-/-*^ cells on days 4, 7, and 10. (B) Dot plot showing top 3 marker genes in each cluster. (C) Temporal profiles of the proportions of 6 clusters. (D) Violin plots depicting the expression levels of neuronal genes in neuronal clusters. (E) GSEA of more highly (red) or lowly (blue) represented genes in *Per1*^*-/-*^ macrophages than in WT and *Per2*^*-/-*^ macrophages on day 7. (F) Heatmaps of the genes in the TNFα and IL-6 signaling pathways up-regulated in *Per1*^*-/-*^ cells in (E). (G) Heatmap of axon guidance genes common to the top and bottom panels in (E).* *p* < 0.05 and ** *p* < 0.01 with exact negative binomial test for differential gene expression. *n* = 1 for each sample. GSEA, Gene Set Enrichment Analysis; iN, induced neuron; scRNA-seq, single-cell RNA sequencing; UMAP, uniform manifold approximation and projection; WT, wild-type.

To explore potential cellular diversity during iN reprogramming, we performed single-cell RNA sequencing (scRNA-seq) of cells on days 4, 7, and 10 of the 3 genotypes. We captured 10,623 cells on average from each sample and obtained 72,767 average reads per cell, which allowed us to analyze 4,418 genes per cell at median. Uniform manifold approximation and projection (UMAP) identified 6 cell type clusters, including neurons, fibroblasts, and macrophages (Figs 2A, 2B, and S3A). The most abundant population was fibroblasts that were not reprogrammed, occupying up to 71% of the total cells depending on the day (Fig 2C). This was followed by gradually decreased neurons and increased macrophages over the culture period. Note that neurons were defined by a set of marker genes, not by βIII tubulin or MAP2 alone. The expression levels of BAM (including the transduced and endogenous transcripts) and 3 neuronal markers (*Tubb3* for βIII tubulin, *Mapt* for Tau, and *Map2*) in each cell were similar between the 3 genotypes each day except for lower levels of some genes in WT neurons on day 10 (Fig 2D).

We anticipated that many neuronal genes were expressed at lower levels in *Per1*^*-/-*^ neurons than in WT and *Per2*^*-/-*^ neurons. However, Gene Set Enrichment Analysis (GSEA) of the neuronal clusters did not reveal *Per1*^*-/-*^ cell-specific down-regulation of neuronal genes compared with other genotypes, except for down-regulation of neuronal system genes in comparison to *Per2*^*-/-*^ neurons on days 7 and 10 (S3B Fig, blue). We also noted up-regulation of genes related to inflammatory cytokines (response to INFα, INFγ, and TNFα) in *Per1*^*-/-*^ neurons (S3B Fig, red), which could correspond to the up-regulation of inflammatory genes on days 2 and 4 found in the bulk RNA-seq. The neuronal cluster could be divided into 11 subclusters based on gene signatures but the division did not correspond to the expression of any specific neurotransmitters (S3C and S3D Fig). Similarly, the macrophage cluster was segregated into 8 subclusters but they were not related to the pro-inflammatory M1 or anti-inflammatory M2 polarization (activation) (S4A and S4B Fig); thus, the subclustering was not informative to dissect the differences between the 3 genotypes.

GSEA of days 4 and 7 macrophages revealed overrepresentation of inflammatory pathway genes related to IFNα, IFNγ, TNFα, IL-6, IL-10, and chemokines in *Per1*^*-/-*^ cells, which disappeared on day 10, indicating that the activation of the inflammatory genes was a temporary event (Figs 2E, 2F, and S4C). Another notable finding was the lower expression of axon guidance genes in *Per1*^*-/-*^ macrophages than in macrophages of other genotypes on all 3 days. The detected 63 axon guidance genes covered several central gene families in this category, such as the families of Ephrins (*Efn*) and their receptors (*Eph*), Netrins (*Ntn*) and their receptors Dcc and Unc5s, Semaphorins (*Sema*) and their receptors Plexins (*Plxn*), Slits (*Slit*) and their receptors Robos (*Robo*), and Slit-Robo Rho GTPase Activating Proteins (*Srgap*) (Fig 2G). Macrophages are known to express these proteins but the net result of the loss of these proteins would be complex since some of them attract while others repel growth cones depending on the context [18]. In summary, macrophages could have 2 functions—inflammatory activation and axon guidance—during iN reprogramming.

### Macrophages inhibit iN reprogramming

**Fig 3:**
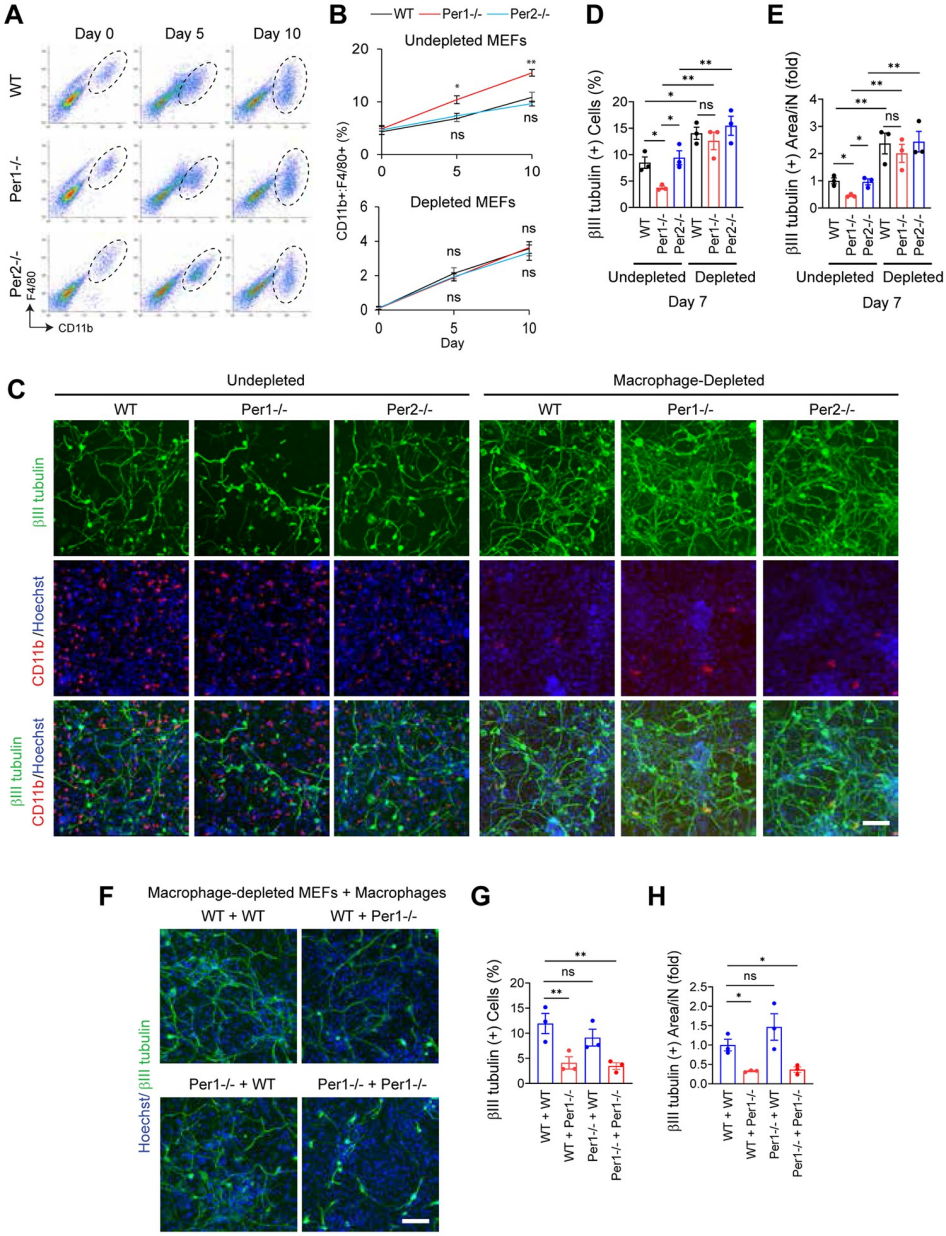
Inhibition of iN reprogramming by macrophages. (A) Flow cytometry to quantify macrophage fractions with CD11b and F4/80 antibodies. (B) Temporal profiles of macrophage fractions in undepleted (top) and macrophage-depleted (bottom) MEFs during iN reprogramming. (C) Immunofluorescence staining of iNs with antibodies against βIII tubulin and CD11b on day 7. (D) Percentage of βIII tubulin (+) cells related to (C). (E) βIII tubulin (+) neurite area in each βIII tubulin (+) cell related to (C). The value with undepleted WT cells was defined as 1.0. (F) Immunofluorescence staining of iNs with βIII tubulin antibody on day 7 after mixing macrophage-depleted MEFs (before “+”) and macrophages (after “+”) of the 2 genotypes each. (G) Percentage of βIII tubulin (+) cells related to (F). (H) βIII tubulin (+) neurite area in each βIII tubulin (+) cell related to (F). * *p* < 0.05 and ** *p* < 0.01 with ordinary one-way ANOVA with Bonferroni’s multiple comparison test; ns indicates statistically not significant. All data were based on biological triplicates with technical triplicates each. The data underlying this figure can be found in S1 Data. iN, induced neuron; MEF, mouse embryonic fibroblast; WT, wild-type.

We next focused on the roles of macrophages in iN reprogramming. Flow cytometry with 2 surface markers CD11b and F4/80 revealed a 2- to 3-fold increase in the macrophage fraction from around 5% to 10%–15% over the course of 10 days of reprogramming in all 3 genotypes (Fig 3A and 3B, top). The higher increase of macrophages in the *Per1*^*-/-*^ culture than in the cultures of other genotypes (6% higher on day 10) might contribute to the lower efficiency of reprogramming. The increase in the macrophage fraction could be due to maturation of macrophages and/or proliferation (see below for more details).

To understand whether macrophages inhibited or promoted iN reprogramming, we immunologically depleted them from MEFs to less than 1% before making iNs. The macrophage fraction still increased during reprogramming after depletion but remained <4% (Fig 3B, bottom and S5A Fig). The depletion increased the number of iNs (defined by the expression of βIII tubulin or MAP2) and the neurite area per iN in all 3 genotypes. Most notably, the number of *Per1*^*-/-*^ iNs was increased by 2- to 3-fold and the neurite area per iN by 5- to 8-fold depending on the neuronal markers, making them indistinguishable from WT iNs (Figs 3C–3E and S5B–S5D). This is evidence of an inhibitory effect of macrophages of all genotypes. As a side note, some macrophages were closely associated with neurites but functional consequences of this association remain unknown (Fig 3C).

In a complementary study, we added back WT or *Per1*^*-/-*^ macrophages with >95% purity (S5E and S5F Fig) to macrophage-depleted MEFs of each genotype at the 1:9 ratio for the reprogramming assay. *Per1*^*-/-*^ macrophages decreased iNs and neurites regardless of the genotypes of the depleted MEF partner compared with WT macrophages (Figs 3F–3H and S5G–S5I). On the other hand, the genotype of the depleted MEFs did not affect the reprogramming efficiency. In summary, iN reprogramming was inhibited by contaminating macrophages in all genotypes and this inhibitory effect was amplified by *Per1* depletion.

Next, we revisited the increase in the macrophage fractions during the reprogramming (Fig 3B). This time we incubated the cells with EdU for 24 h to detect slowly proliferating macrophages (doubling time of >5 days for *Per1*^*-/-*^ macrophages based on Fig 3B, top). This longer incubation revealed EdU uptake in up to 5% of the cells until day 10; there was no statistically significant difference between each genotype (S6A Fig). However, co-staining of EdU and CD11b showed that macrophages represented a higher fraction within the EdU (+) population in the *Per1*^*-/-*^ culture compared with other genotypes on day 10 (S6B and S6C Fig). Related to this, a higher fraction of *Per1*^*-/-*^ macrophages incorporated EdU in comparison to other genotypes on the same day (S6D Fig). Thus, proliferation appeared to have contributed to the increased macrophage fractions in all 3 genotypes during the reprogramming, which was promoted by *Per1* depletion.

Despite the slow cell proliferation, the total cell number did not change during iN reprogramming (S1F Fig). To understand the reason for this discrepancy, we applied TUNEL staining and found that up to 2% of the cells were undergoing apoptosis in all 3 genotypes (S6E and S6F Fig, undepleted). The balance between cell proliferation and apoptosis appeared to have maintained the constant cell number in the culture. We next tested whether *Per1*^*-/-*^ non-macrophage population contained more apoptotic cells than WT and *Per2*^*-/-*^ counterparts to explain the similar total cell number across all 3 genotypes despite more active proliferation of *Per1*^*-/-*^ macrophage (S1F Fig). However, there was no statistically significant difference in the TUNEL (+) fractions between undepleted and macrophage-depleted MEFs in all 3 genotypes during the reprogramming (S6F Fig). It was possible that the <6% difference in the abundance of macrophages between the *Per1*^*-/-*^ culture and the rest (Fig 3B, top, day 10) was too subtle to make a detectable difference in the total cell number. We also attempted to quantify apoptotic non-macrophage cells by co-staining of TUNEL and CD11b but the TUNEL staining quenched the CD11b signal, precluding the double staining.

### MEFs support macrophage proliferation and maturation as feeder cells

**Fig 4:**
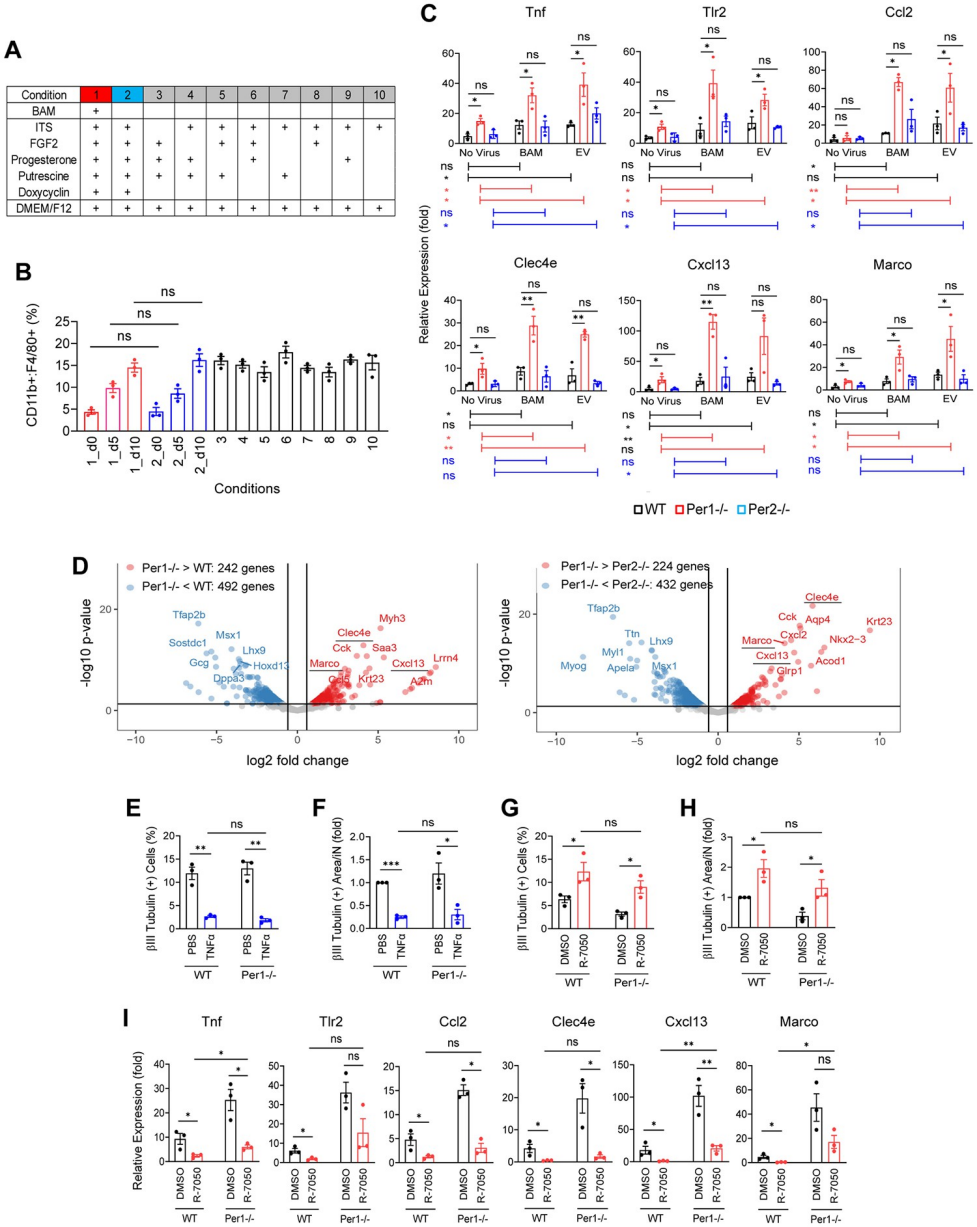
Analyses of the increased macrophage fractions and up-regulation of macrophage genes. (A) A list of the components in the N3 medium selectively used to culture MEFs under 10 conditions. (B) Macrophage fractions after culture of *Per1*^*-/-*^ MEFs under the conditions in (A) for the indicated days (conditions 1 and 2) and 10 days (conditions 3–10). There was no statistically significant difference between condition 1 on day 10, condition 2 on day 10, and conditions 3 to 10. (C) PCR of TNFα pathway genes (top) and other inflammatory genes (bottom) on day 7 after culture without virus, with BAM viruses, and with EV virus. The values with WT cells without virus on day 0 were defined as 1.0. (D) Volcano plots of scRNA-seq data on day 7. The underlined 3 genes were studied in (C). (E) Fractions of βIII tubulin (+) cells on day 10 after culture with 2 ng/ml TNFα. (F) βIII tubulin (+) area per iN in (E). The value with PBS and WT cells was defined as 1.0. (G) Fractions of βIII tubulin (+) cells on day 10 after culture with 100 nM R-7050. (H) βIII tubulin (+) area per iN in (G). The value with DMSO and WT cells was defined as 1.0. (I) PCR of TNFα pathway genes and inflammatory genes. * *p* < 0.05 and ** *p* < 0.01 with ordinary one-way ANOVA with Bonferroni’s multiple comparison test (C) or with two-tailed *t* test (B and E–I); ns indicates statistically not significant. All data were based on biological triplicates with technical triplicates each. The data underlying this figure can be found in S1 Data. EV, empty vector; iN, induced neuron; MEF, mouse embryonic fibroblast; scRNA-seq, single-cell RNA sequencing; WT, wild-type.

Although macrophage fractions increased by up to 2-fold during the initial 5 days of iN reprograming, some macrophage genes were up-regulated more than 2-fold in the bulk RNA-seq analysis during this period, suggesting that the cells were undergoing maturation as well (S2E Fig versus Fig 3B). We investigated several candidates that caused an increase in the macrophage fractions and up-regulation of macrophage genes. The first candidate was the BAM viruses but the presence or absence of the viruses did not affect macrophage fractions in *Per1*^*-/-*^ cells (Fig 4A and 4B, condition 1 versus 2). Five macrophage marker genes were up-regulated on days 5 and 10 compared with day 0 in all 3 genotypes but the levels of up-regulation were also indistinguishable between the presence and absence of the viruses (S7A Fig). Second, the well-known macrophage activator endotoxin was undetectable (<0.01 EU/ml) in the culture medium with ELISA on days 0, 5, and 10 in the 3 genotypes. Third, complement in the fetal bovine serum (FBS) could also have activated macrophages but MEFs were cultured with heat-inactivated serum from the beginning; serum was not included in the N3 medium during iN reprogramming. Fourth, we systematically depleted each component from the N3 medium without BAM and applied the cells to flow cytometry and PCR (Fig 4A, conditions 3 to 10). Macrophage fraction was not affected by any of the depletions (Fig 4B, conditions 3 to 10 and S7B Fig). Additionally, up-regulation levels of some macrophage genes were not changed by the depletions (S7C Fig); thus, N3 components were not responsible for macrophage proliferation or maturation.

Finally, we tested whether non-macrophage cells in the MEF culture stimulated macrophages serving as feeder cells. Indeed, purified macrophages did not proliferate (S7D Fig) or up-regulate selected genes when cultured in the N3 medium without BAM for 9 days (S7E Fig). Mouse microglia reach complete maturation by 1 month after birth and their survival and maturation are dependent on M-CSF (a main driver for macrophage proliferation and differentiation), IL-34, and other cytokines secreted by neurons, microglia, and astrocytes [19]. The mRNA level of *Il34* was very low (<15 counts per million reads or CPM) with the scRNA-seq analysis. ELISA detected <2 ng/ml M-CSF in the supernatant between day 0 and 10 in all genotypes (S7F Fig) but this concentration range was not sufficient to induce proliferation (S7G Fig) or maturation of purified macrophages (S7H Fig). Combined effects of these and other cytokines are likely to contribute to macrophage proliferation and maturation.

During these studies, we also found that an addition of FBS to the N3 medium masked maturation and an increase in the macrophage fractions. While total cell numbers increased, macrophage fractions remained low in the presence of FBS (S8A and S8B Fig). Up-regulation of 5 macrophage genes was also undetectable in the presence of FBS (S8C Fig). These results could be due to more preferential proliferation of non-macrophages cells and/or active suppression of macrophages by FBS. These findings could explain why maturation and proliferation of macrophages were not evident during culture in DMEM with 10% FBS prior to the iN reprogramming assay.

### Autocrine TNFα activates macrophages inhibiting iN reprogramming

To understand why *Per1*^*-/-*^ macrophage were more inhibitory than other macrophages, we focused on the genes up- or down-regulated specifically in *Per1*^*-/-*^ macrophages in the scRNA-seq data. They included up-regulated genes in the IL-6, IL-10, IFNα, IFNγ, and TNFα pathways (Figs 2E and S4C). Expression levels of these cytokine genes were very low (<3 CPM) across all cell types except for *Tnf* (encoding TNFα), which was almost exclusively expressed in macrophages in scRNA-seq (S1 Table). TNFα, one of the best characterized autocrine inflammatory activators of macrophages, and 2 downstream genes *Tlr2* (Toll like receptor 2) and *Ccl2* (C-C motif chemokine ligand 2) were up-regulated without virus and more highly with BAM or empty virus on day 7 compared with day 0 (Fig 4C). The virus-induced up-regulation was more prominent with *Per1*^*-/-*^ cells than others. Moreover, 3 inflammatory genes particularly highly up-regulated in *Per1*^*-/-*^ macrophages (*Clec4e*, *Cxcl13*, and *Marco*) in scRNA-seq exhibited a similar trend (Fig 4C and 4D).

The TNFα concentration in the culture supernatant was <2 ng/ml during iN programming with *Per1*^*-/-*^ MEFs (S8D Fig). When we added 2 ng/ml TNFα to the reprogramming assay with macrophage-depleted MEFs of WT and *Per1*^*-/-*^, the number of iNs decreased and neurite development was severely inhibited regardless of the genotype (Fig 4E and 4F). This finding indicated that TNFα could substitute for the inhibitory role of macrophages. In a complementary study, we inhibited TNFα by adding the receptor antagonist R-7050 to undepleted MEFs because knockdown by shRNA was inefficient with macrophages. The inhibitor increased iNs and neurite development with both genotype cells, making WT and *Per1*^*-/-*^ iNs similar (Fig 4G and 4H). The inhibitor also down-regulated the TNFα-related genes and inflammatory genes although some genes remained more highly expressed in *Per1*^*-/-*^ cells than in WT cells (Fig 4I). The numbers of total cells and the fractions of macrophages were not affected by these treatments (S8E and S8F Fig). These results highlight TNFα as a major player for the macrophage-induced inhibition of iN reprogramming with both WT and *Per1*^*-/-*^ MEFs. The stronger inhibition by *Per1*^*-/-*^ macrophages could be due to the higher expression level of *Tnf* but additional inhibitor(s) secreted by macrophages could also be contributing. For example, the decreased axon guidance proteins mentioned above could be playing additional roles.

**Fig 5:**
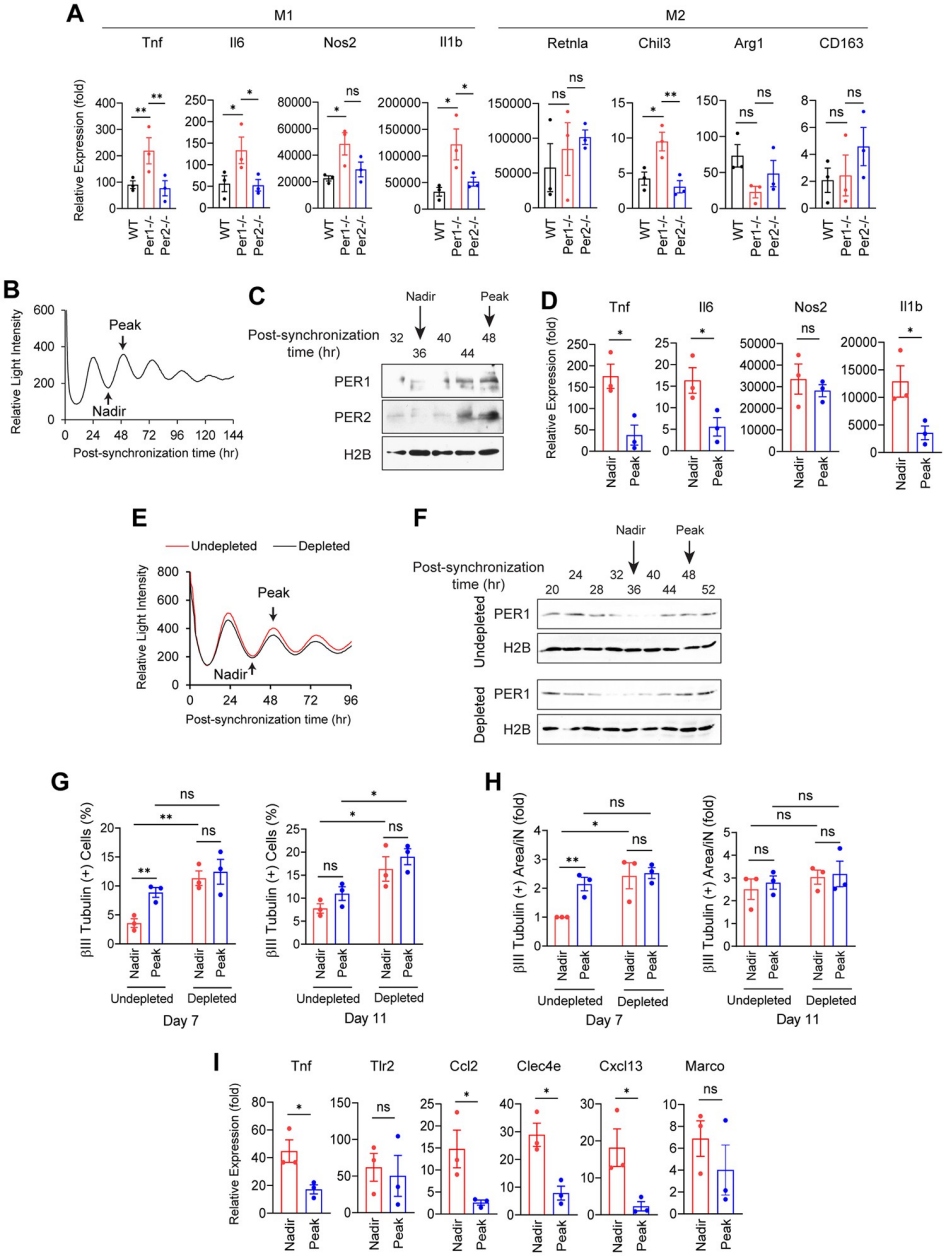
Hypersensitivity of macrophages caused by *Per1* depletion. (A) PCR of marker genes for M1 and M2 polarizations of macrophages. The value with WT cells without stimulations was defied as 1.0. The M1 polarization was induced by INFγ for 12 h followed by LPS for 4 h; M2 polarization by IL-4 for 24 h. (B) Luciferase activity of *Per2*::*Luc* macrophages after synchronization with dexamethasone between -1 and 0 h. (C) Western blotting demonstrating the expression of PER1 and PER2 in synchronized macrophages. Histone H2B was used as the loading control. (D) PCR of M1 marker genes after an addition of IFNγ at the nadir or peak in (B) followed by LPS. (E) Luciferase activity of *Per2*::*Luc* MEFs after synchronization with dexamethasone between -1 and 0 h. (F) Western blotting showing the expression of PER1 in synchronized MEFs. (G) Fractions of βIII tubulin (+) cells. BAM viruses were added for 4 h starting at the nadir or peak in (E). (H) βIII tubulin (+) neurite area in each βIII tubulin (+) cell related to (G). The value with day 7 nadir with undepleted cells was defined as 1.0. (I) PCR of inflammatory genes on day 7. * *p* < 0.05 and ** *p* < 0.01 with ordinary one-way ANOVA with Bonferroni’s multiple comparison test (A) or with two-tailed *t* test (D and G–I); ns indicates statistically not significant. All data were based on biological triplicates with technical triplicates each. The data underlying this figure can be found in S1 Data. LPS, lipopolysaccharide; MEF, mouse embryonic fibroblast; WT, wild-type.

To understand whether the higher maturation of *Per1*^*-/-*^ macrophages induced by the feeder cells reflected general hypersensitivity of the cells, we induced polarization of purified macrophages into the M1 and M2 states comparing the 3 genotypes. Macrophages are commonly polarized into the M1 or M2 state by IFNγ and lipopolysaccharide (LPS) or by IL-4, respectively, although many intermediate states coexist in reality [20]. This experiment revealed that *Per1*^*-/-*^ macrophages were more prone to be polarized to the M1 state than WT and *Per2*^*-/-*^ cells as demonstrated by higher up-regulation of 4 marker genes, whereas marker genes for the M2 state did not show a consistent trend (Fig 5A). Thus, the higher maturation by the feeder cells, the stronger responses to the viruses, and the more prominent M1 polarization by the inflammatory stimuli all appear to reflect a general hypersensitive state of macrophages created by *Per1* depletion. As a reference, expression levels of the 8 polarization markers, except for *tnf*, were all very low (<2 CPM) in the scRNA-seq data, suggesting that macrophages were not polarized into typical M1 or M2 states during iN reprogramming.

### The efficiency of iN reprogramming depends on circadian timing

Daily oscillations of the *Per1* level suggested daily fluctuations in macrophages’ sensitivity. To test this, we purified macrophages from MEFs of *Per2*::*Luc* mice that had the luciferase gene knocked into the C terminus at the *Per2* locus as a fusion gene [21] and synchronized circadian rhythms with dexamethasone for 1 h. The luciferase activity exhibited circadian oscillations as expected (Fig 5B). Western blotting verified fluctuations of the PER1 and PER2 protein levels that corresponded to the luciferase activity levels (Fig 5C). Three M1 marker genes were more highly activated when IFNγ was added while the luciferase level was at the nadir compared with the addition at the peak (Fig 5D).

Extending this observation to iN reprogramming, we transduced BAM for 4 h to synchronized *Per2*::*Luc* MEFs at the nadir or peak of the luciferase activity and compared the reprograming efficiency 7 (168 h) and 10 (240 h) days later (Fig 5E). The oscillations of the PER1 protein level in undepleted and macrophage-depleted MEFs were confirmed by western blotting (Fig 5F). There were more iNs with more developed neurites in the peak transduction than in the nadir transduction on day 7 but the differences faded away by day 11 with undepleted MEFs (Fig 5G and 5H, undepleted). Similarly, some inflammatory genes were more highly up-regulated by the nadir transduction than by the peak transduction on day 7 with undepleted MEFs (Fig 5I). The circadian timing-dependent responses of macrophages were consistent with the circadian fluctuations in macrophage sensitivity. Similar circadian timing-dependent differences in cell reactivities have been reported by us and others in muscle regeneration and skin wound healing models [17,22].

Next, we repeated the nadir and peak transductions with macrophage-depleted MEFs to understand whether reprogramming efficiency of non-macrophage cells was also dependent on the circadian timing of transduction (Fig 5E and 5F, depleted). However, there was no difference in iN numbers or neurite development between nadir and peak transductions (Fig 5G and 5H, depleted). Thus, the circadian effect on the iN reprogramming was primarily mediated by macrophages, not fibroblasts or other cells. This result was consistent with the similar reprogramming efficiency between WT and *Per1*^*-/-*^ MEFs once macrophages were depleted (Figs 3D and 3E, and S5C and S5D).

### Macrophages inhibit iPSC reprogramming

We selected iPS cell (iPSC) formation as another serum-free reprogramming model to test macrophages’ proliferation and maturation as well as inhibitory roles. We transduced *M*_*3*_*O*, *Sox2*, *Klf4*, and *c-Myc* into MEFs that harbored the *Egfp* gene as a fusion gene with *Oct4* (*Oct4-Egfp* MEFs) as described before [23]. *M*_*3*_*O* was a fusion gene between *Oct4* and the transactivation domain of *MyoD*, which facilitated iPSC reprogramming. In the previous protocol [23], we subcultured the transduced cells at a low cell density (<1,000 cells/cm^2^) on feeder cells to accurately count EGFP (+) iPSC colonies. However, this was <3.3% of the cell density used in iN reprogramming (3 × 10^4^ cells/cm^2^), making macrophages’ effects undetectable. This was probably a reason why the roles of macrophages remained unnoticed. Unknown influences by feeder cells—irradiated MEFs including macrophages—would also complicate the interpretation. On the other hand, if we skip the subculture and maintain a high cell density after transduction, too many colonies make it difficult to count the colonies after day 8. To address this dilemma, we focused on the number of emerging iPSC colonies between days 4 and 8 without subculture.

**Fig 6:**
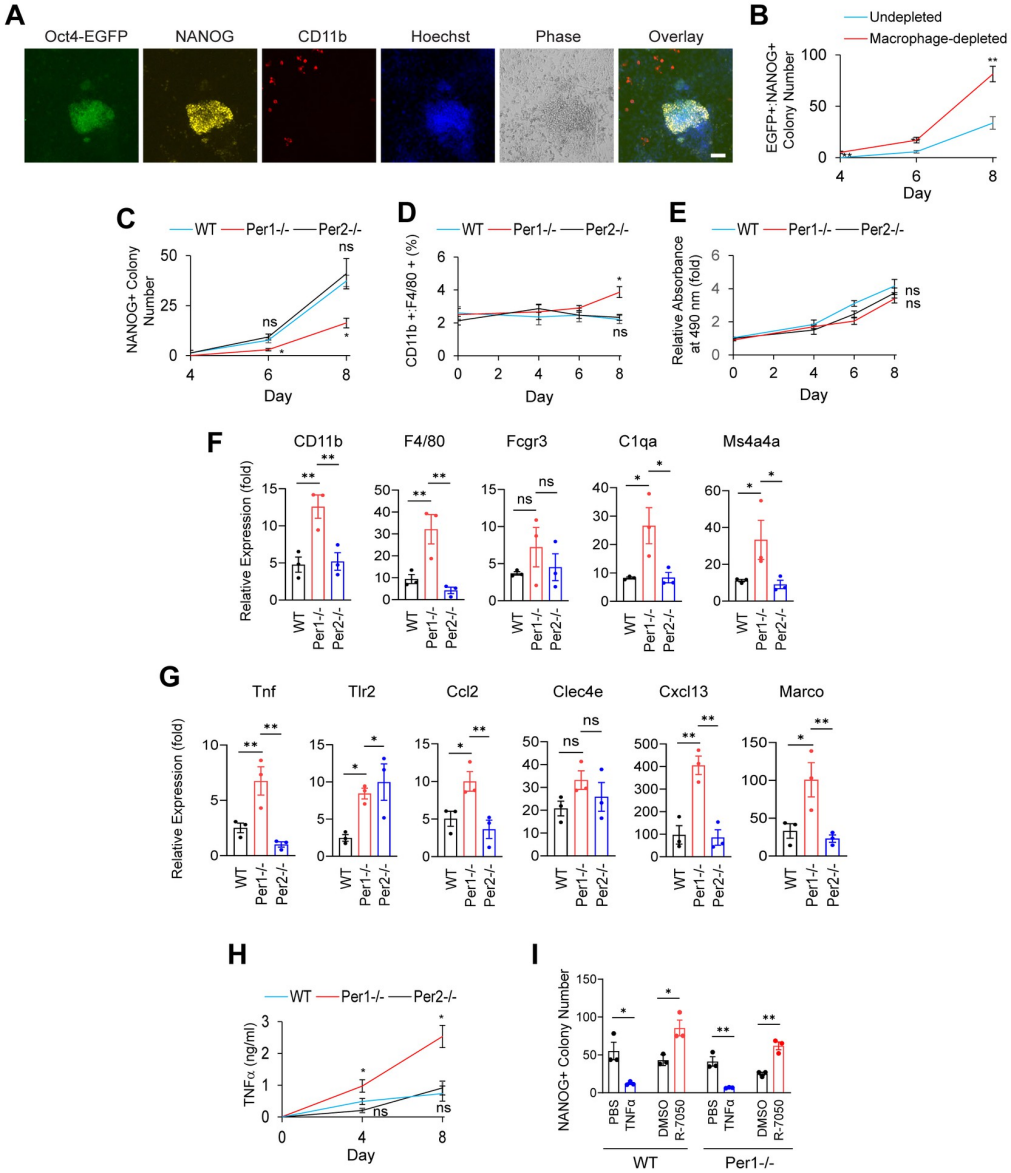
Inhibition of iPSC reprogramming by macrophages. (A) Immunofluorescence staining of an iPSC colony. GFP was detected with antibody due to quenching by paraformaldehyde fixation. Bar, 100 μm. (B) Temporal profiles of the numbers of GFP+:NANOG+ iPSC colonies per 1.3 × 10^4^ cells in a well of a 48-well plate, comparing undepleted and macrophage-depleted Oct4-GFP MEFs. (C) Temporal profiles of the numbers of NANOG+ colonies comparing 3 genotypes. (D) Macrophage fractions during iPSC reprogramming. (E) Temporal profiles of the numbers of total cells in each well with an MTS assay. (F) PCR of macrophage marker genes on day 8 during iPSC reprogramming. The value with WT cells on day 0 without virus was defined as 1.0. (G) PCR of the TNFα pathway genes and inflammatory genes on day 8. The value with WT cells on day 0 without virus was defined as 1.0. (H) ELISA of TNFα in the supernatant of iPSC culture. The culture medium was not changed for 48 h before harvest for ELISA. (I) Numbers of iPSC colonies on day 8 in the continuous presence of the indicated chemicals. PBS and TNFα were added to macrophage-depleted MEFs, whereas DMSO and R-7050 were added to undepleted MEFs.* *p* < 0.05 and ** *p* < 0.01 with ordinary one-way ANOVA with Bonferroni’s multiple comparison test (C–H) or with two-tailed *t* test (B and I); ns indicates statistically not significant. All data are based on biological triplicates with technical triplicates each. The data underlying this figure can be found in S1 Data. iPSC, induced pluripotent stem cell; MEF, mouse embryonic fibroblast; WT, wild-type.

iPSC colonies, defined by double positivity for EGFP and NANOG (Fig 6A), in macrophage-depleted wells appeared 1 or 2 days earlier and the number was more than 2-fold on day 8 compared with those in undepleted wells (Fig 6B). When we compared the number of iPSC colonies between WT, *Per1*^*-/-*^, and *Per2*^*-/-*^ MEFs using NANOG as a maker, the number with *Per1*^*-/-*^ MEFs was around 40% of the rest on day 8 (Fig 6C). Although the macrophage fraction did not substantially increase in any genotypes of MEFs by day 8 unlike in iN reprogramming, total cell numbers increased due to rapid proliferation of partially reprogrammed iPSCs, causing a net increase in the number of macrophages, in particular *Per1*^*-/-*^ macrophages (Fig 6D and 6E). Additionally, *Per1*^*-/-*^ cells showed higher up-regulation of macrophage markers and inflammatory genes than others (Fig 6F and 6G). ELISA indicated that the TNFα concentration in the culture medium was <3 ng/ml during iPSC reprograming (Fig 6H). An addition of TNFα and R-7050 decreased and increased iPSC colonies, respectively (Fig 6I), consistently with the role of TNFα as an inhibitor of iPSC reprogramming. Thus, the roles of macrophages were similar between iN and iPSC reprogramming.

## Discussion

**Fig 7:**
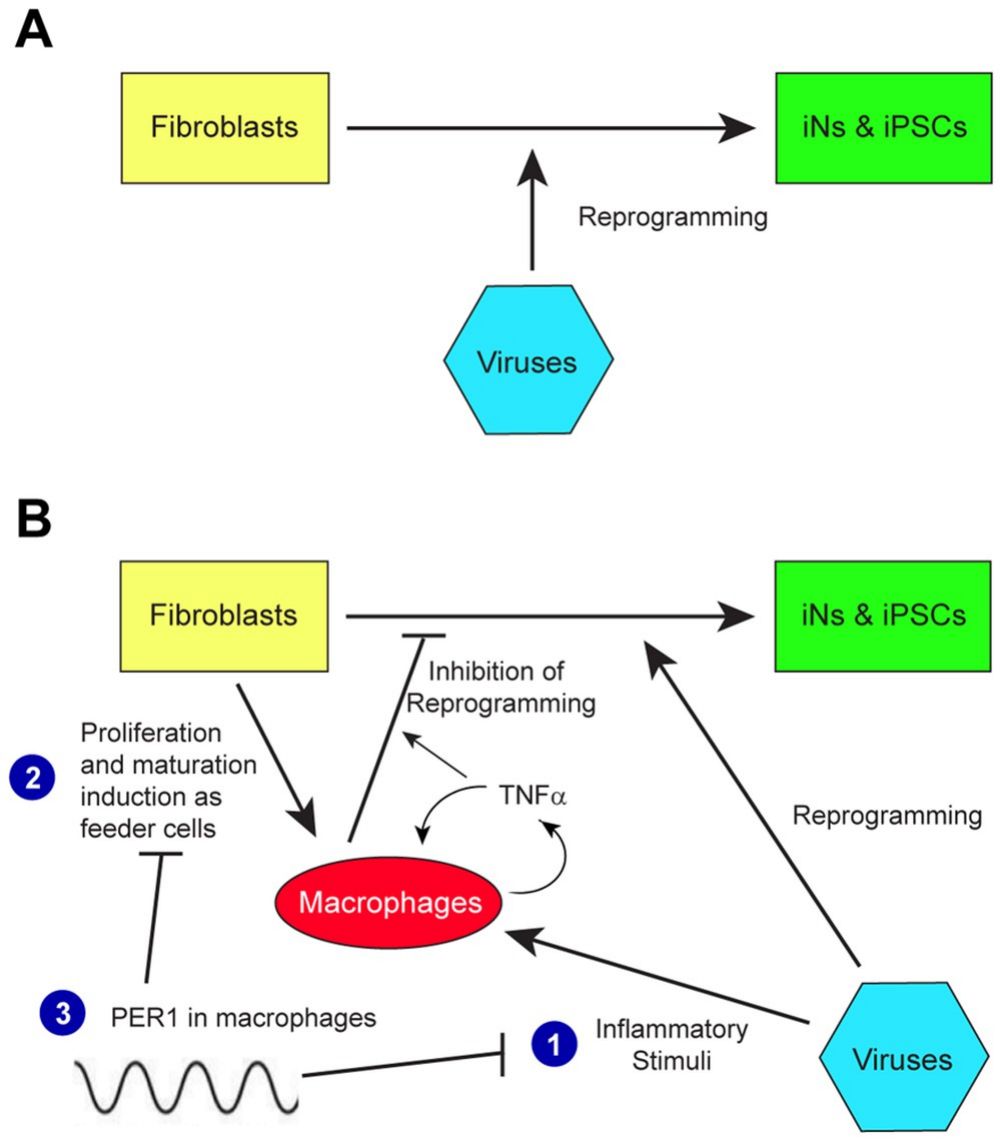
Summary of the findings. (A) Current focus in the reprogramming study. (B) Our findings. See text for explanation. iN, induced neuron; iPSC, induced pluripotent stem cell.

Our findings can be summarized as follows. Prevailing research on cell reprogramming has been almost exclusively focused on the process of how target cells, such as fibroblasts, are reprogrammed by transduced viruses and other agents (Fig 7A). However, we found that coexisting macrophages in the MEF culture play hidden roles through 3 mechanisms (Fig 7B). First, transduced viruses trigger inflammatory responses from macrophages and promote secretion of cytokines and chemokines such as TNFα, which inhibit iN and iPSC reprogramming. TNFα further stimulates macrophages in an autocrine manner. Second, proliferation and maturation of embryonic macrophages are stimulated by non-macrophage cells serving as feeders independently of viruses, which also leads to TNFα up-regulation in macrophages. Third, these 2 mechanisms are repressed by PER1 in WT macrophages; however, genetic depletion or circadian down-regulation of PER1 de-repress and hyperactivates macrophages, unmasking the inhibitory roles of macrophages. It remains unknown whether circadian down-regulation of PER1 is the only reason why WT macrophages inhibited iN and iPSC reprogramming.

This study was intended to find a direct link between circadian rhythms and the cells undergoing reprogramming (MEFs) but these 2 turned out to be indirectly linked via macrophages. Among the three-way links between cell reprogramming, circadian rhythms, and macrophages, the circadian rhythm-macrophage link is the most established one [24–26]. Around 8% to 16% of all mRNAs and 29% of all proteins display circadian expression in macrophages, including many inflammatory genes and proteins [27,28]. In addition, secretion levels of TNFα and IL-6 from macrophages depend on the time-of-day of the stimulation by LPS in vitro. Furthermore, transcription of the LPS downstream components is regulated by circadian rhythms at multiple levels, including the regulators of LPS binding to TLR4, homodimerization of TLR4, the MAPK pathway, and controllers of the downstream effectors NF-κB and AP-1. Finally, double disruption of *Cry1*/*Cry2* or *Per1*/*Per2* and a single depletion of Rev-Erbα make bone marrow-derived macrophages more reactive to inflammatory stimulations [29–33]. Thus, circadian regulation is a tightly integrated component in the macrophage reactivity, which was reflected in the *Per1*^*-/-*^ macrophages in our results.

We know almost nothing about the second interaction: the reprogramming-circadian rhythm link. Nonetheless, many examples of circadian regulation of cell differentiation [14,15] suggest the presence of such a link. We also know little about the reprogramming-macrophage (or other neutral-looking bystander cell) interactions. However, when we extend this to the neuron–macrophage interactions as a reference, some potentially relevant examples are known. In physiological examples, microglia play crucial roles in building neuronal networks during development through multiple mechanisms [34]. For example, they modulate the number of neuronal progenitor cells by actively eliminating apoptotic cells. They also constantly survey synaptic activity and eliminate less active synapses (activity-dependent synaptic pruning) by phagocytosis, supporting establishment of proper neuronal circuitry [35]. In a pathological example, microglia promote tissue damage in experimental autoimmune encephalomyelitis by secreting pro-inflammatory cytokines, such as TNFα, IL-6, and GM-CSF, at the peak of inflammation [19]. Later in the healing phase, they support resolution of the inflammation by secreting anti-inflammatory cytokines, including TGFβ and IL-10, and by clearing myelin debris via phagocytosis. Time-laps imaging of the interactions between iNs and macrophages could reveal direct interactions between them during reprogramming.

When a peripheral nerve is severed, Schwann cells and macrophages form a glial bridge connecting the proximal and distal nerve ends as a guidance for regenerating axons [18,36]. Crosstalk between the axon guidance molecules mentioned earlier ensures proper reconnections of the severed axons. For example, the glycoprotein Slit3 secreted from macrophages interacts with the Robo1 receptor on the surface of Schwann cells. The Ephrin-Eph receptor signaling links fibroblasts and Schwann cells. Several guidance proteins also function as pro-inflammatory (Sema4B and EphA2) and anti-inflammatory (Slit2 and Netrin1) ques. There might be disorganized crosstalk between macrophages, fibroblasts, and oligodendrocytes during iN reprogramming.

Our work highlights the importance of considering hidden roles of immune cells in reprogramming. Although we only assessed the efficiency of the reprogramming in the current study, immune cells could also affect the reprogramming process, such as transcription, epigenetics, and metabolism, inside the target cells as the mechanisms underlying the inhibition. The interactions between the target cells and immune cells would play more prominent and vital roles in in vivo reprogramming. This is especially true when reprogramming is applied to treat injuries, such as ischemic heart diseases and spinal cord injuries recruiting plentiful immune cells [37,38]. In our case, repeated subculture of MEFs eliminated floating immune cells, including lymphocytes and neutrophils. However, these cells are abundantly present in the damaged tissues and almost all types of immune cells are under control of circadian rhythms [39,40].

Organoid culture is another context where our findings could become relevant. Different populations of immune cells have been incorporated into brain, gut, and other organoids to recreate more physiologically relevant tissue models [41,42]. These immune cells are likely to behave under circadian control although rhythmicity might be undetectable unless artificially synchronized. By extension, organoid models of tumor immunology could also be affected by circadian rhythms [43]. Our results send precautions to the communities studying all these research areas.

## Materials and methods

### Mouse strains

All protocols were approved by the Institutional Animal Care and Usage Committee of the University of Minnesota (2111-39606A). *Per1*^*+/-*^ mice (B6.129-*Per1*^*tm1Drw*^/J, 010491), *Per2*^*+/-*^ mice (B6.129-*Per2*^*tm1Drw*^/J, 010492), *Per2*::*Luc2* mice (B6.129S6*-Per2*^*tm1Jt*^/J, 006852), and Oct4-GFP mice (B6.129S4*-Pou5f1*^*tm2Jae*^/J, 008214) were purchased from Jackson Laboratory. *Per1*^*-/-*^, *Per2*^*-/-*^, and WT mice were obtained by breeding and identified by genotyping according to Jackson Laboratory protocols. Mice were monitored by the Research Animal Resources staff of the University of Minnesota in specific pathogen-free housing. Mice were given standard chow and access to drinking water without restrictions. Mice were euthanized via CO_2_ inhalation. All methods align with the Panel of Euthanasia of the American Veterinary Medical Association recommendations.

### Preparation of mouse embryonic fibroblasts (MEFs)

All FBS used in the current work was heat-inactivated by incubation in a water bath at 56°C for 30 min. MEFs were prepared from day 13.5 embryos as follows after removing the head and the heart. Embryos were minced with a scalpel in 0.25% trypsin and 0.53 mM EDTA for 3 min. After an addition of 10% FBS to inhibit trypsin, the cell suspension was centrifuged at 200 x*g* for 5 min. Supernatant prepared from 4 embryos was cultured in 10% FBS in DMEM in a 15 cm dish coated with 0.1% gelatin. When the cells reached 80% confluent 2 to 3 days later, they were harvested and frozen as P0 (passage 0) in 90% FBS and 10% DMSO and stored in liquid nitrogen for future use. All MEFs were used at P1. Sex of the embryos was not determined.

### Preparation of iNs

This was based on the original BAM protocol [2]. On day -6, 293FT cells (Thermo Fisher Scientific, R70007) were seeded in DMEM with 10% tetracyclin-negative FBS (Tc (-) FBS hereafter) at 3 × 10^5^ cells/well in a 12-well plate. On day -5, cells were transfected with 0.17 μg each of Tet-O-FUW-Ascl1 (Addgene, 27150), Tet-O-FUW-Brn2 (27151), and Tet-O-FUW-Myt1 (27152) along with 0.2 μg each of pCMV-VSV-G (8454), pRSV-Rev (12253), and pMDLg/pRRE (12251) using 2.75 μl Lipofectamine 2000 (Thermo Fisher Scientific, 11668019) to prepare the BAM virus. In another well, 0.5 μg FUW-M2rtTA (Addgene, 20342) was transfected along with 0.2 μg each of pCMV-VSV-G, pRSV-Rev, and pMDLg/pRRE to prepare the M2 virus. The culture medium was replaced with fresh DMEM with 10% Tc (-) FBS 5 h later. On day -4, MEFs were seeded at 1.2 × 10^5^ cells/well in 12-well plates in DMEM with 10% Tc (-) FBS. On day -3, culture supernatant of the transfected cells was applied to a 0.45 μm syringe filter and added to MEFs as follows: 400 μl BAM virus, 400 μl M2 virus, 800 μl DMEM with 10% Tc (-) FBS, and 2 μg/ml polybrene (MilliporeSigma, H9268). On day -2, the medium was replaced with DMEM with 10% Tc (-) FBS. On day -1, cells were subcultured into a 48-well plate at 3 × 10^4^ cells/well in DMEM with 10% Tc (-) FBS. The wells had been successively coated at 4°C with 20 μg/ml poly-D lysine (MP Biomedical, 0215017510) and 10 μg/ml laminin (Thermo Fisher Scientific, 23017–015) for 16 h each prior to use. On day 0, medium was replaced with the N3 medium (1× insulin, transferrin, and selenium [ITS, Thermo Fisher Scientific, 414100–045], 0.2 μm progesterone [MilliporeSigma, P8783], 0.1 μm putrescine dihydrochloride [MilliporeSigma, P5780], and 10 ng/ml basic FGF [Peprotech, 450–33] in DMEM/F12) with 2 μg/ml doxycycline hyclate (MP Biomedical, 198955) after wash with PBS twice. The medium was replaced every 2 days thereafter. For ELISA, culture medium was not changed for 48 h prior to medium harvest. The concentration of LPS in the medium was determined with a ToxiSensor chromogenic LAL endotoxin assay kit (GenScript, L00350Y). PBS was used as control for 2 ng/ml TNFα (Peprotech, 315-01A); DMSO was used as control for 100 nM R-7050 (Cayman, 16870) to test the role of TNFα. A TNFα uncoated ELISA kit (Thermo Fisher Scientific, 88-7324-22) and an M-CSF quantikine ELISA kit (R&D Systems, MMC00B) were used to determine the concentrations of TNFα and M-CSH, respectively. The cell number in each well was assessed with a CellTiter 96 AQ_ueous_ One Solution Cell Proliferation Assay (MTS, Promega, G3582).

The efficiency of iN reprogramming was quantified with 2 parameters. The first one was βIII tubulin (+) cell % or MAP2 (+) cell % calculated as follows. The total number of nuclei (denominator) stained with Hoechst 33342 in each image captured with a 10× objective was counted with the Fiji program (https://imagej.net/software/fiji/downloads). The number of nuclei (numerator) embedded in the cytoplasm expressing βIII tubulin or MAP2 was manually counted. The numerator was divided by the denominator to obtain βIII tubulin (+) cell % or MAP2 (+) cell %. Second, neurite area (μm^2^), which was defined as the area covered by the βIII tubulin or MAP2 signal, was measured with Fiji and divided by the number of βIII tubulin (+) or MAP2 (+) cells in each image. This value was normalized against the control sample in each experiment. Three biologically independent experiments with 3 different images each were used to obtain mean ± SEM for both parameters.

### Preparation of iPSCs

This was based on our previous work [23]. On day -4, Plat-E cells [44] were seeded at 3 × 10^5^ cells/3.5 cm dish. On day -3, 0.4 μg each of pMXs-mM_3_O-IP (Addgene, 46644), pMXs-Sox2-IP (15919), pMXs-Klf4-IP (15920), and pMXs-c-Myc-IP (15921) were transfected into Plat-E cells with 4.5 μl FuGENE 6 (Promega, E2691). mM_3_O is a fusion gene in which the first 62 amino acids in the transactivation domain of the mouse *MyoD* gene were fused to the amino terminus of mouse *Oct4* cDNA to promote the iPSC reprogramming [23]. On day -2, MEFs were seeded at 5 × 10^4^ cells/well of a 12-well plate in DMEM with 10% FBS. On day -1, supernatant of the transfected Plat-E cells was filtered through a 0.45 μm syringe filter and added to the MEFs along with 10 μg/ml polybrene. On day 0, medium was replaced with iPSC medium (DMEM, 20% KnockOut serum replacement [Thermo Fisher Scientific, 10828028], 100 μm MEM nonessential amino acids, 55 μm 2-mercaptoethanol, 2 mM L-glutamine, and 1,000 u/ml leukemia inhibitory factor [LIF, MilliporeSigma, ESG1106]). The medium was changed every 2 days thereafter.

### Flow cytometry

MEFs were sequentially stained with Ghost Dye 780 (1 μl, Tonbo, 13–0865 T100), Fc blocker (1 μg, BD Pharmingen, 553141), and CD11b-PE (0.12 μg, Miltenyi Biotec, 130-113-806, RRID:AB_ 2751172) and F4/80-APC (0.3 μg, Miltenyi Biotec, 130-116-525, RRID:AB_2733417) prior to loading to a Fortessa LSRII H4760 Cell Analyzer (BD Biosciences). The number in the parenthesis indicates the used amount of each reagent per 1 × 10^6^ cells. Floreada.io (https://floreada.io/analysis) was used to analyze data and make figures.

### Purification of macrophages from MEF culture

WT and *Per1*^*-/-*^ MEFs were stained as described above and CD11b^+^ cells were separated with anti-PE MicroBeads (Miltenyi Biotec, 130-048-801), MS columns (130-042-201), and a MiniMACS separator (130-042-102). Purity of CD11b (+) cells in the bound and unbound fractions was determined with flow cytometry; each fraction was used in the iN reprogramming assay in different combinations. To induce the M1 polarization, macrophages were cultured with 10 ng/ml IFNγ (Peprotech, 315–05) for 12 h, followed by 100 ng/ml LPS (InvivoGen, tlrl-3pelps) for 4 h before harvest for PCR. For the M2 polarization, macrophages were cultured with 100 ng/ml IL-4 (Peprotech, 214–14) for 24 h.

### Circadian synchronization

Macrophages prepared from *Per2*::*Luc* MEFs were cultured in 10% FBS and 25 ng/ml M-CSF (Shenandoah, 100–03) in αMEM in a 35 mm dish. When the cells became 70% confluent, the cells were treated with 0.2 μm dexamethasone (MilliporeSigma, D4902) for 1 h for circadian synchronization followed by washing with PBS twice. Fresh culture medium of 10% FBS, 25 ng/ml M-CSF, 1 mM D-luciferin (Gold Biotechnology, 103404-75-7), and 25 mM HEPES (pH 7.8) in αMEM was added to the dish. The dish was sealed with parafilm to prevent medium evaporation and placed in a LumiCycle 32 luminometer (Actimetrics) in a 37°C incubator for recording of the luciferase activity. Synchronized cells were induced to polarize into the M1 state by an addition of IFNγ when the luciferase activity was at the peak or nadir, followed by LPS as described above. The cells were cultured without D-luciferin outside the LumiCycle 32 once INFγ was added. The same synchronization procedure was applied to MEFs for iN reprogramming in DMEM with 10% FBS. The BAM viruses were transduced for 4 h when the luciferase activity was at the peak or nadir. BAM expression was induced with doxycycline on the next day without subculture.

### Immunofluorescence staining of cells

Cells were fixed with 4% paraformaldehyde for 10 min and permeabilized with 0.5% Triton X100 in PBS for 5 min. Following a blocking step with 10% FBS and 0.2% Tween 20 in PBS for 10 min, the cells were stained with a primary antibody at 25°C for 1 h and then incubated with a fluorescence-labeled secondary antibody at 25°C for 1 h. DNA was counterstained with Hoechst 33342 (MilliporeSigma, B2261). Following primary antibodies were used: βIII tubulin (abcam, ab18207, RRID:AB_44319), MAP2 (Cell Signaling Technology, 4542, RRID:AB_10693782), NANOG (R&D Systems, AF2729, RRID:AB_2150103), and GFP (Santa Cruz Biotechnology, sc-9996, RRID:AB_627695). Following secondary antibodies were used: Alexa Fluor 488 goat anti-mouse IgG (Thermo Fisher Scientific, A-11029, RRID:AB_2534088), Alexa Fluor 488 goat anti-rabbit IgG (Thermo Fisher Scientific, A-11034, RRID: AB_2576217), and Alexa Fluor 647 chicken anti-goat IgG (Thermo Fisher Scientific, A-21469, RRID: AB_2535872). To assess cell proliferation, cells were pulsed with 1 μg/ml of EdU for 6 h and stained with a Click-iT EdU kit Alexa Fluor 488 (Thermo Fisher Scientific, C10337). TUNEL (+) apoptotic cells were detected with an In Situ Cell Death Detection Kit, Fluorescein (Roche, 11684795910). The frequency of EdU (+) or TUNEL (+) cells was determined by dividing the number of signal (+) cells by the total number of Hoechst 33342 (+) nuclei in each image, all counted with the Fiji program. Three biologically independent experiments with 3 different images each (containing 1,300 to 3,200 nuclei each) were used to obtain mean ± SEM. Fluorescence images were captured using MetaMorph Basic software ver. 7.8.12.0 (Molecular Devices) with UPlanFLN 10× objective lens 0.31 Ph1 (Olympus), LUCPlan FLN 20× objective lens 0.45 Ph1 (Olympus), and a C11440 digital camera (Hamamatsu) attached to an IX73P2F microscope (Olympus). The images were processed with Adobe Photoshop and Illustrator CS6 (https://helpx.adobe.com/creative-suite.html).

### Quantitative RT-PCR (qRT-PCR)

RNA was extracted from cells using a Quick RNA Microprep (Zymo Research, R1051) and purity was assessed using a microvolume spectrophotometer (DeNovix, DS-11 FX+). cDNA was synthesized with ProtoScript II Reverse Transcriptase (New England Biolabs, M0368L). qPCR was performed with the primers listed in S2 Table and qPCRBIO SyGreen Blue Mix Lo-ROX (Genesee Scientific, 17-505B) in a Mastercycler realplex^2^ thermocycler (Eppendorf). PCR conditions were as follows: initial denaturation at 95°C for 2 min, 40 cycles of 95°C for 5 s, 60°C for 30 s, and 72°C for 30 s, and a melting curve step to check the specificity of the reaction. mRNA expression levels were analyzed by normalizing expression values to glyceraldehyde 3-phosphate dehydrogenase (*Gapdh*) expression. Mean ± SEM of biological triplicates with technical triplicates each were calculated.

### Western blotting

Western blotting was done as previously described [17]. Specifically, whole-cell extracts obtained from 2 × 10^5^ cells with an NE-PER Nuclear and Cytoplasmic Extraction kit (Thermo Fisher Scientific, 78833) were loaded into a 12% SDS-PAGE gel. After completion of electrophoresis, the proteins were transferred to an Immobilon P membrane (EMD Millipore, IPVH00010) at 25°C overnight. The next day, the membrane was blocked with 5% non-fat dry milk (BioRad, 180171A) in PBT (0.2% Tween 20 in PBS) for 1 h at 25°C. Proteins were then labeled with the primary antibody diluted in 5% milk in PBT at 25°C for 1 h. After washing with PBT for 5 min 3 times, the membranes were incubated with secondary antibody in 5% milk in PBT for 1 h at 25°C. After washing the membrane with PBT 6 times, the chemiluminescence signal was detected with a SuperSignal West Dura kit (Thermo Fisher Scientific, 34075) and X-ray films or an iBright Imaging System (Thermo Fisher Scientific). Following antibodies were used: PER1 (MilliporeSigma, AB2201, RRID:AB_1587378), PER2 (NOVUS, NB-100-125, RRID_AB_10000765), histone H2B (Thermo Fisher Scientific, MA5-14835, RRID:AB_10982286), goat anti-mouse IgG-HRP (Bio-Rad, 170–6516, RRID:AB_11125547), and goat anti-rabbit IgG-HRP (Bio-Rad, 170–6515, RRID:AB_11125142).

### RNA-seq: Library construction, quality control, and sequencing

Total RNA was prepared from iN culture before reprogramming (day 0), and days 2 and 4 during reprogramming. RNA concentration and RNA integrity number (RIN) were measured with an Agilent BioAnalyzer 2100. RIN was 10 for all RNA samples. mRNA was purified from total RNA using poly-T oligo-attached magnetic beads. After fragmentation, the first strand cDNA was synthesized using random hexamer primers, followed by the second strand cDNA synthesis for nondirectional library. Library preparation was completed by end repair, A-tailing, adapter ligation, size selection, amplification, and purification. The library was checked with a Qubit and real-time PCR for quantification and a Bioanalyzer for size distribution. Clustering of the index-coded samples was performed according to Illumina’s instructions. The libraries were sequenced on an Illumina platform and paired-end reads were generated.

### RNA-seq: Data analysis

Raw reads of the fastq format were processed through in-house perl scripts. In this step, clean reads were obtained by removing low-quality reads and the reads containing adaptors or poly-N from raw data. At the same time, Q20, Q30, and GC content in the clean data were calculated. All the downstream analyses were based on clean data with high quality. Paired-end clean reads were aligned to the reference genome Mus musculus GRCm38 (ftp://ftp.ensembl.org/pub/release-94/fasta/mus_musculus/dna/Mus_musculus.GRCm38.dna.primary_assembly.fa.gz and ftp://ftp.ensembl.org/pub/release-94/gtf/mus_musculus/Mus_musculus.GRCm38.94.gtf.gz) using Hisat2 v2.0.5 (https://daehwankimlab.github.io/hisat2/). featureCounts v1.5.0-p3 (http://subread.sourceforge.net/) was used to count read numbers mapped to each gene. Fragments per kilobase of exon per million mapped fragments (FPKM) of each gene was calculated based on the length of the gene and read counts mapped to the gene. Differential expression analysis was performed using the DESeq2 R package (1.20.0) (https://www.r-project.org/). Genes with log2 fold change ≥0.58 (≥1.5-fold) and an adjusted *p*-value ≤0.05 were assigned as differentially expressed.

Enrichment of specific gene pathways in differentially expressed genes were identified by applying the clusterProfiler R package (https://www.r-project.org/) to the databases of Gene Ontology (http://www.geneontology.org), KEGG (http://www.genome.jp/kegg/), Reactome (https://reactome.org/), Disease Ontology (http://disease-ontology.org), and DisGeNET (http://www.disgenet.org). Adjusted *p*-value ≤0.05 were considered significantly enriched. The GSEA analysis tool (http://www.broadinstitute.org/gsea/index.jsp) was used along with the databases of Gene Ontology, KEGG, Reactome, Disease Ontology, and DisGeNET.

### scRNA-seq: From single-cell capture to sequencing

The cells were counted using an acridine orange/propidium iodide exclusion count assay with a LUNA-FL Dual Fluorescence Cell counter (Logos Biosystems). A single-cell suspension of 17,000 cells was loaded onto a Chromium Next GEM Chip G (10X Chromium, PN-1000127) aiming at capturing 10,000 cells. Chromium Next GEM Single Cell 3′ Kit v3.1 (10X Chromium, PN-1000268) was used on a Chromium Controller to generate Gel Beads in Emulsion (GEM) and add barcodes following the manufacturer’s instructions. After reverse transcription and amplification of cDNA, quality and quantity of cDNA was assessed with an Agilent Tapestation High Sensitivity D5000 ScreenTape (Agilent, 5067-5592). Fragmentation, end repair, A-tailing, size selection, adaptor ligation, PCR for library construction, and post-library quality control were all performed following the instruction of the kit. The libraries were sequenced with an Illumina NovaSeq S4 in a 150 paired-end reads format with a minimum sequencing target of 25,000 reads per cell. The resulting data was processed using Cell Ranger v7.1 (https://support.10xgenomics.com/single-cell-gene-expression/software/pipelines/latest/what-is-cell-ranger).

### scRNA-seq: Data analysis

scRNA-seq data were analyzed using R and Bioconductor packages (https://www.bioconductor.org/). The basic quality metrics including unique molecular identifiers (UMIs) per cell and gene counts per cell were estimated using the scater package (packages/release/bioc/html/scater.html). Cells with low library size, low numbers of features, or a high number of mitochondrial genes were filtered out using the quickPerCellQC function in scater package. The cutoff for UMI is 3,000 and mitochondrial genes is 20%. Batch effects were removed, and datasets from each sample were integrated using the fastMNN method in the bachelor R package (packages/release/bioc/html/batchelor.html). Hypervariable expressed genes were identified by the getTopHVGs function in the scran package (packages/release/bioc/html/scran.html). The top 500 genes with the highest biological variances were selected for dimension reduction using the UMAP algorithm. Clusters were identified using the buildSNNGraph function in the scran package. Marker genes for the clusters were determined by combined pairwise comparisons (*t* tests) using the findMarkers function of the scran package. Significant genes were selected by a log2 fold change ≥1 and combined *p*-value ≤0.05. Functional annotation and overrepresentation analysis of these gene sets was done by goana function in the limma package (packages/release/bioc/html/limma.html). The gene differential analysis of the same cluster/cell between different sample/group was performed using the edgeR package (packages/release/bioc/html/edgeR.html).

### Statistical analysis

Following statistical programs were used based on the assumption of normal distributions of the values although most of the experiments were based on only biological triplicates. Ordinary one-way ANOVA with Bonferroni’s multiple comparison test was used to compare 3 samples (WT, *Per1*^*-/-*^, and *Per2*^*-/-*^ cells) to determine the statistical significance of the difference in the iN numbers, neurite areas, EdU uptake, qRT-PCR data, and others. Two-tailed *t* test was used to compare 2 samples (e.g., nadir versus peak, undepleted versus depleted, and others) in these studies. The mean + or ± SEM obtained from biological triplicates with technical triplicates was shown in each graph unless stated otherwise.

## Supporting information

S1 Fig Immunostaining of MAP2 and cell proliferation during iN reprogramming. (A) Western blotting of WT, *Per1*^*-/-*^, and *Per2*^*-/-*^ MEFs with PER1 and PER2 antibodies. Histone H2B was used as a loading control. (B) Immunofluorescence staining of iNs with MAP2 antibody on day 11. DNA was counterstained with Hoechst 33342. Bar, 100 μm. (C) Percentages of MAP2 (+) cells. (D) MAP2 (+) neurite area in each MAP2 (+) cell. The value with day 7 WT cells was defined as 1.0. (E) EdU uptake in WT cells. MEFs without virus were used as positive control. (F) MTS assay representing total cell numbers at each time point during iN reprogramming. The value of WT cells on day 0 was defined as 1.0. There was no statistically significant difference between the 3 genotypes each day or between day 0 and day 9 for each genotype. * *p* < 0.05 and ** *p* < 0.01 with ordinary one-way ANOVA with Bonferroni’s multiple comparison test; ns indicates statistically not significant. All data were based on biological triplicates with technical triplicates each. The data underlying this figure can be found in S1 Data.
(TIF)

S2 Fig Transcriptomic analyses of iNs prepared from WT, *Per1*^*-/-*^, and *Per2*^*-/-*^ MEFs. (A) Heatmap of differentially expressed genes comparing the 3 genotypes on 3 days. (B) Principal component analysis of the RNA-seq data. (C) Heatmap of the 80 genes that were commonly up-regulated in *Per1*^*-/-*^ cells compared with WT and *Per2*^*-/-*^ cells on days 2 and 4. (D) David pathway analysis of the 80 genes. (E) Temporal profiles of the expression levels of selected macrophage gene in the 80 genes based on the RNA-seq data. * *p* < 0.05 and ** *p* < 0.01 with ordinary one-way ANOVA with Bonferroni’s multiple comparison test; ns indicates statistically not significant. All data were based on biological triplicates. The data underlying this figure can be found in S1 Data.
(TIF)

S3 Fig scRNA-seq analyses of iNs and other cells. (A) UMAP comparing 6 clusters between 3 genotypes on 3 days. (B) GSEA of more highly (red) or lowly (blue) represented genes in *Per1*^*-/-*^ neurons than in other genotypes on days 4, 7, and 10. The gene sets were not enriched in the comparison pairs where they are not shown. (C) UMAP of neuronal subclusters. All 9 samples were combined in (C) and (D). (D) Dot plot of neuronal subclusters. *n* = 1 for each sample.
(TIF)

S4 Fig scRNA-seq analyses of macrophages. (A) UMAP of macrophage subclusters. All 9 samples were combined in (A) and (B). (B) Dot plot of macrophage subclusters. (C) GSEA of more highly (red) or lowly (blue) represented genes in *Per1*^*-/-*^ macrophages than in other genotypes on days 4 and 10. *n* = 1 for each sample.
(TIF)

S5 Fig Inhibition of iN reprogramming by macrophages. (A) Flow cytometry to quantify the macrophage fractions in macrophage-depleted MEFs during iN reprogramming. (B) Immunofluorescence staining of iNs with MAP2 antibody on day 11. (C) Percentages of MAP2 (+) cells related to (B). (D) MAP2 (+) neurite area in each MAP2 (+) cell related to (B). The value with undepleted WT cells was defined as 1.0. (E) Flow cytometry to quantify purified macrophage fractions. (F) Percentages of purified macrophages on day 0. (G) Immunofluorescence staining of iNs with MAP2 antibody on day 11 after mixing macrophage-depleted MEFs (before “+”) and macrophages (after “+”) of 2 genotypes each. (H) Percentages of MAP2 (+) cells related to (G). (I) MAP2 (+) neurite area in each MAP2 (+) cell related to (G). * *p* < 0.05 and ** *p* < 0.01 with ordinary one-way ANOVA with Bonferroni’s multiple comparison test. (F) Used two-tailed *t* test; ns indicates statistically not significant. All data are based on biological triplicates with technical triplicates each. The data underlying this figure can be found in S1 Data.
(TIF)

S6 Fig Analyses of DNA replication and apoptosis during iN reprogramming. (A) The frequency of EdU (+) cells during iN reprogramming. (B) Representative images of EdU uptake into macrophages (top) and non-macrophages (bottom). (C) The frequency of macrophages within the EdU (+) population during the reprogramming. (D) The frequency of EdU (+) cells within the macrophage population during the reprogramming. (E) TUNEL staining of undepleted WT cells during the reprogramming. (F) The frequency of TUNEL (+) cells in undepleted and macrophage-depleted cells during the reprogramming. There was no statistically significant difference between each genotype on day 10. Bar, 50 μm. * *p* < 0.05 and ** *p* < 0.01 with ordinary one-way ANOVA with Bonferroni’s multiple comparison test. (F) Used two-tailed *t* test; ns indicates statistically not significant. All data were based on biological triplicates with technical triplicates each. The data underlying this figure can be found in S1 Data.
(TIF)

S7 Fig Characterization of macrophage proliferation and maturation during iN reprogramming. (A) PCR of macrophage genes upon culture of MEFs with and without BAM viruses. The values obtained with WT MEFs without virus on day 0 were defined as 1.0. (B) Flow cytometry of *Per1*^*-/-*^ MEFs after culture in N3, N3 without ITS (N3, ITS (-)), and N3 without FGF2, progesterone, and putrescine (N3, FPP (-)) in the absence of viruses. (C) PCR of macrophages genes on day 10 after culture of MEFs under the conditions in (B). (D) MTS assays following the culture of purified macrophages in the N3 medium without BAM viruses. The values with WT cells on day 0 was defined as 1.0. (E) PCR of macrophage marker genes after culture of purified macrophages in the N3 medium without BAM viruses for 9 days. The values obtained with WT macrophages on day 0 were defined as 1.0. (F) ELISA of M-CSF in the supernatant during iN reprogramming. The culture medium was not replaced for 2 days before harvest. (G) MTS assays following the culture of purified macrophages in the N3 medium with 2 ng/ml M-CSF. The values with WT cells on day 0 was defined as 1.0. (H) PCR of macrophage marker genes after culture of purified macrophages in the N3 medium with 2 ng/ml M-CSF for 9 days. The values obtained with WT macrophages on day 0 were defined as 1.0. * *p* < 0.05 and ** *p* < 0.01 with ordinary one-way ANOVA with Bonferroni’s multiple comparison test; ns indicates statistically not significant. All data were based on biological triplicates with technical triplicates each. The data underlying this figure can be found in S1 Data.
(TIF)

S8 Fig The effects of FBS and TNFα on macrophage proliferation and maturation. (A) MTS assays following the culture of WT and *Per1*^*-/-*^ MEFs with and without FBS in the absence of viruses. The values with WT MEFs without FBS was defined as 1.0. (B) Macrophage fractions related to (A). (C) PCR of macrophage genes on day 10 related to (B). (D) ELISA of TNFα in the supernatants during iN reprogramming. (E) MTS assays studying the effects of TNFα and R-7050 on day 10. The values on day 0 were defined as 1.0. (F) The effects of TNFα and R-7050 on the macrophage fractions on day 10. PBS and DMSO were used as controls for TNFα and R-7050, respectively. * *p* < 0.05 and ** *p* < 0.01 with ordinary one-way ANOVA with Bonferroni’s multiple comparison test (A, B, and D) or with two-tailed *t* test (C, E, and F); ns indicates statistically not significant. All data were based on biological triplicates with technical triplicates each. The data underlying this figure can be found in S1 Data.
(TIF)

S1 Table Cytokine values from scRNA-seq data. (XLSX)

S2 Table Sequences of PCR primers. (PDF)

S1 Data Excel data sheet containing raw data and statistical analyses. (XLSX)

S1 Raw Images Uncropped images of western blotting. (PDF)

## Abbreviations

CPM: counts per million
FBS: fetal bovine serum
FPKM: fragments per kilobase of exon per million mapped fragments
GEO: Gene Expression Omnibus
GSEA: Gene Set Enrichment Analysis
iN: induced neuron
iPSC: induced pluripotent stem cell
LPS: lipopolysaccharide
MEF: mouse embryonic fibroblast
RIN: RNA integrity number
scRNA-seq: single-cell RNA sequencing
UMAP: uniform manifold approximation and projection
UMI: unique molecular identifier
WT: wild-type
