# Supplementary figures and images for: The circadian regulator PER1 promotes cell reprogramming by inhibiting inflammatory signaling from macrophages
Source: PLoS Biol. 2023 Dec 4;21(12):e3002419. doi: 10.1371/journal.pbio.3002419 (PMC10721173; doi:10.1371/journal.pbio.3002419)

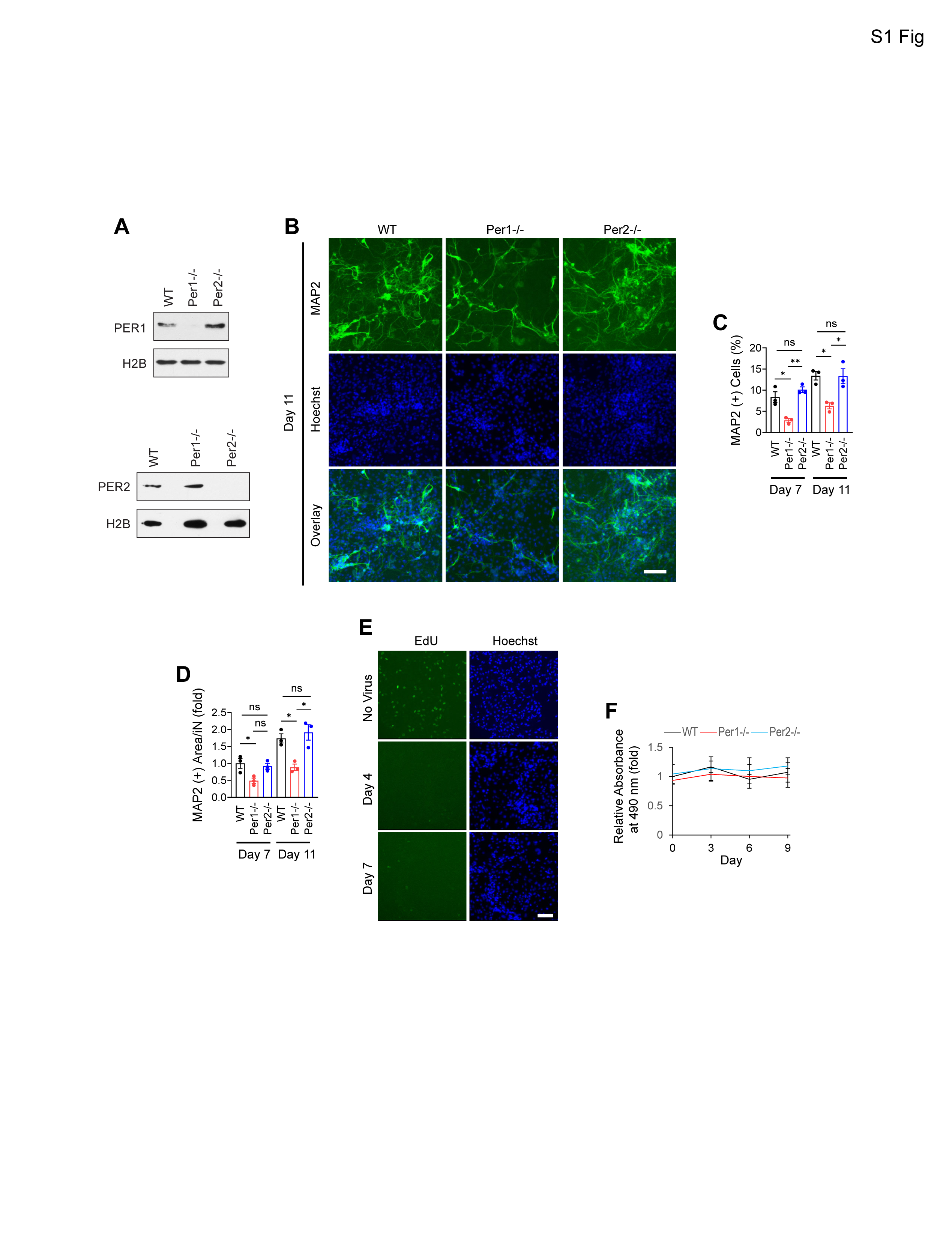

Supplement: S1 Fig — (A) Western blotting of WT, Per1-/-, and Per2-/- MEFs with PER1 and PER2 antibodies. Histone H2B was used as a loading control. (B) Immunofluorescence staining of iNs with MAP2 antibody on day 11. DNA was counterstained with Hoechst 33342. Bar, 100 μm. (C) Percentages of MAP2 (+) cells. (D) MAP2 (+) neurite area in each MAP2 (+) cell. The value with day 7 WT cells was defined as 1.0. (E) EdU uptake in WT cells. MEFs without virus were used as positive control. (F) MTS assay representing total cell numbers at each time point during iN reprogramming. The value of WT cells on day 0 was defined as 1.0. There was no statistically significant difference between the 3 genotypes each day or between day 0 and day 9 for each genotype. * p < 0.05 and ** p < 0.01 with ordinary one-way ANOVA with Bonferroni’s multiple comparison test; ns indicates statistically not significant. All data were based on biological triplicates with technical triplicates each. The data underlying this figure can be found in S1 Data. (TIF) [file pbio.3002419.s001.tif]

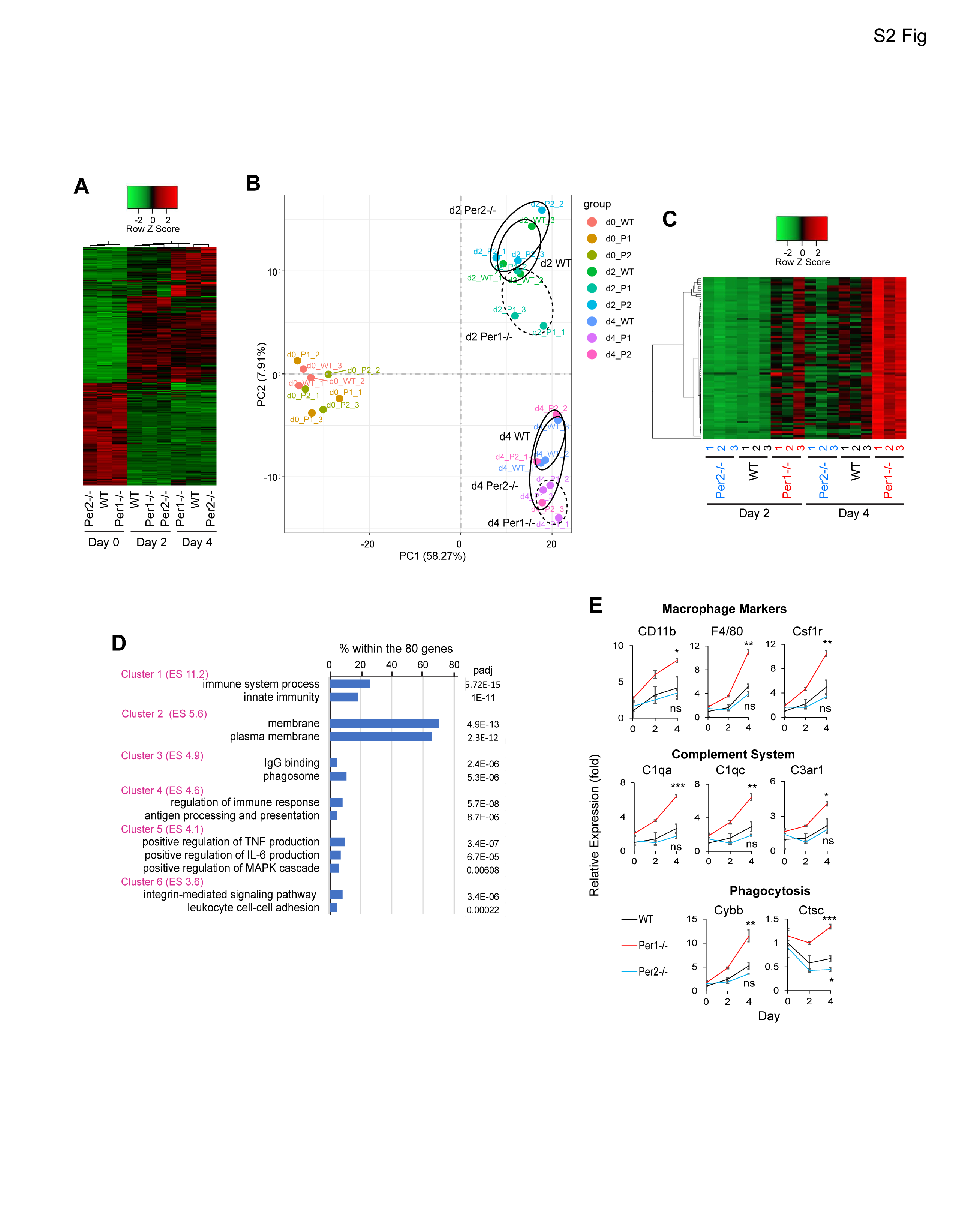

Supplement: S2 Fig — (A) Heatmap of differentially expressed genes comparing the 3 genotypes on 3 days. (B) Principal component analysis of the RNA-seq data. (C) Heatmap of the 80 genes that were commonly up-regulated in Per1-/- cells compared with WT and Per2-/- cells on days 2 and 4. (D) David pathway analysis of the 80 genes. (E) Temporal profiles of the expression levels of selected macrophage gene in the 80 genes based on the RNA-seq data. * p < 0.05 and ** p < 0.01 with ordinary one-way ANOVA with Bonferroni’s multiple comparison test; ns indicates statistically not significant. All data were based on biological triplicates. The data underlying this figure can be found in S1 Data. (TIF) [file pbio.3002419.s002.tif]

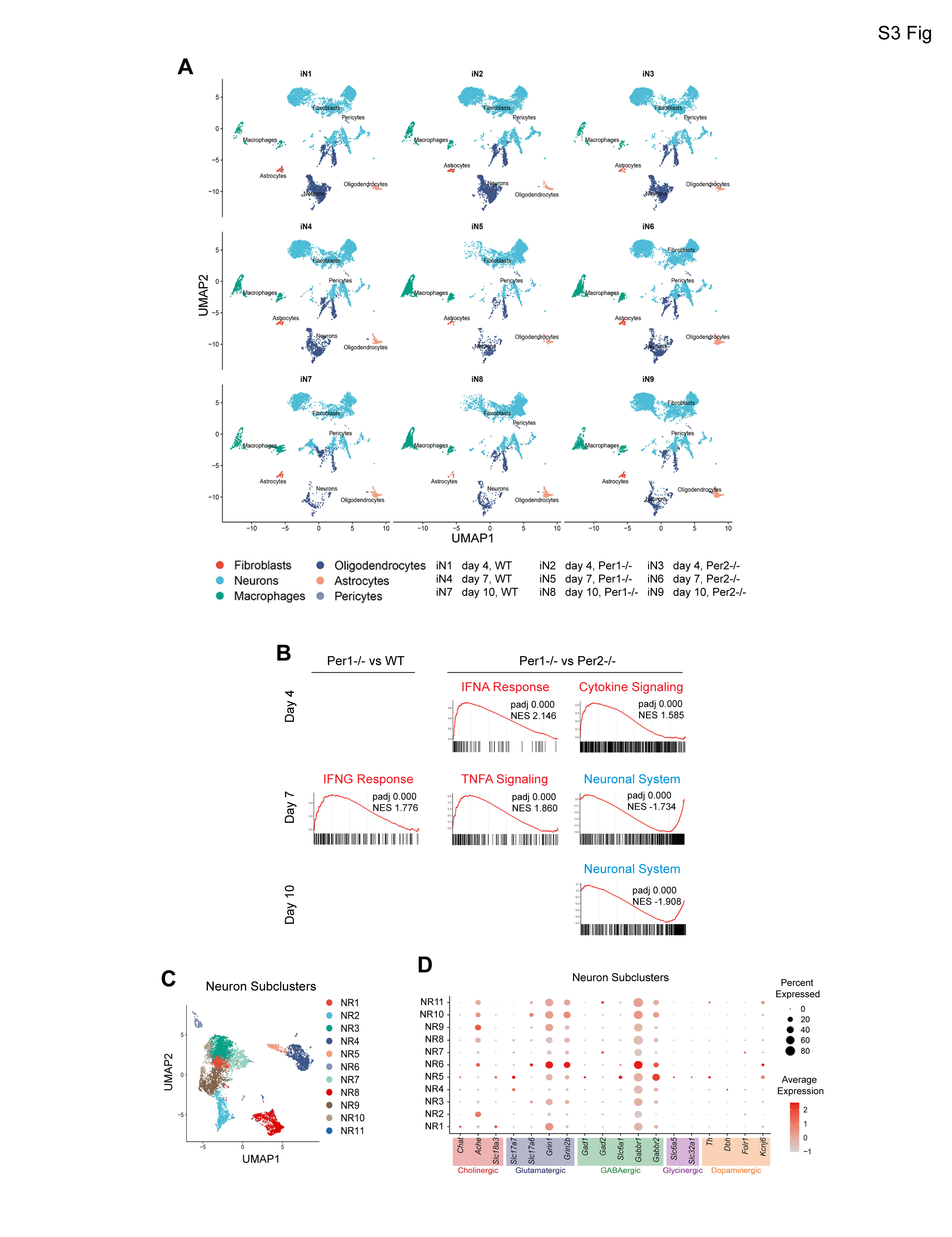

Supplement: S3 Fig — (A) UMAP comparing 6 clusters between 3 genotypes on 3 days. (B) GSEA of more highly (red) or lowly (blue) represented genes in Per1-/- neurons than in other genotypes on days 4, 7, and 10. The gene sets were not enriched in the comparison pairs where they are not shown. (C) UMAP of neuronal subclusters. All 9 samples were combined in (C) and (D). (D) Dot plot of neuronal subclusters. n = 1 for each sample. (TIF) [file pbio.3002419.s003.tif]

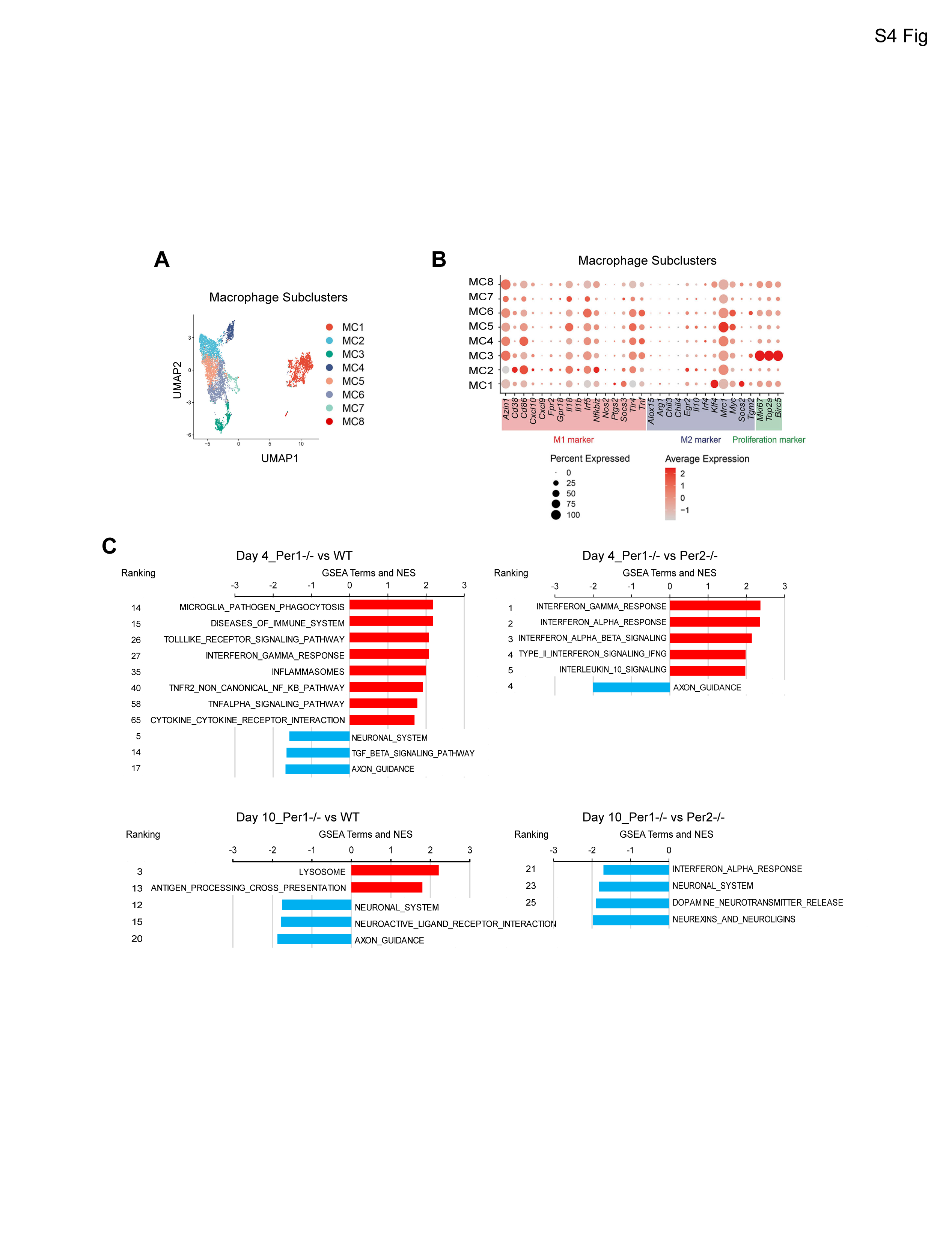

Supplement: S4 Fig — (A) UMAP of macrophage subclusters. All 9 samples were combined in (A) and (B). (B) Dot plot of macrophage subclusters. (C) GSEA of more highly (red) or lowly (blue) represented genes in Per1-/- macrophages than in other genotypes on days 4 and 10. n = 1 for each sample. (TIF) [file pbio.3002419.s004.tif]

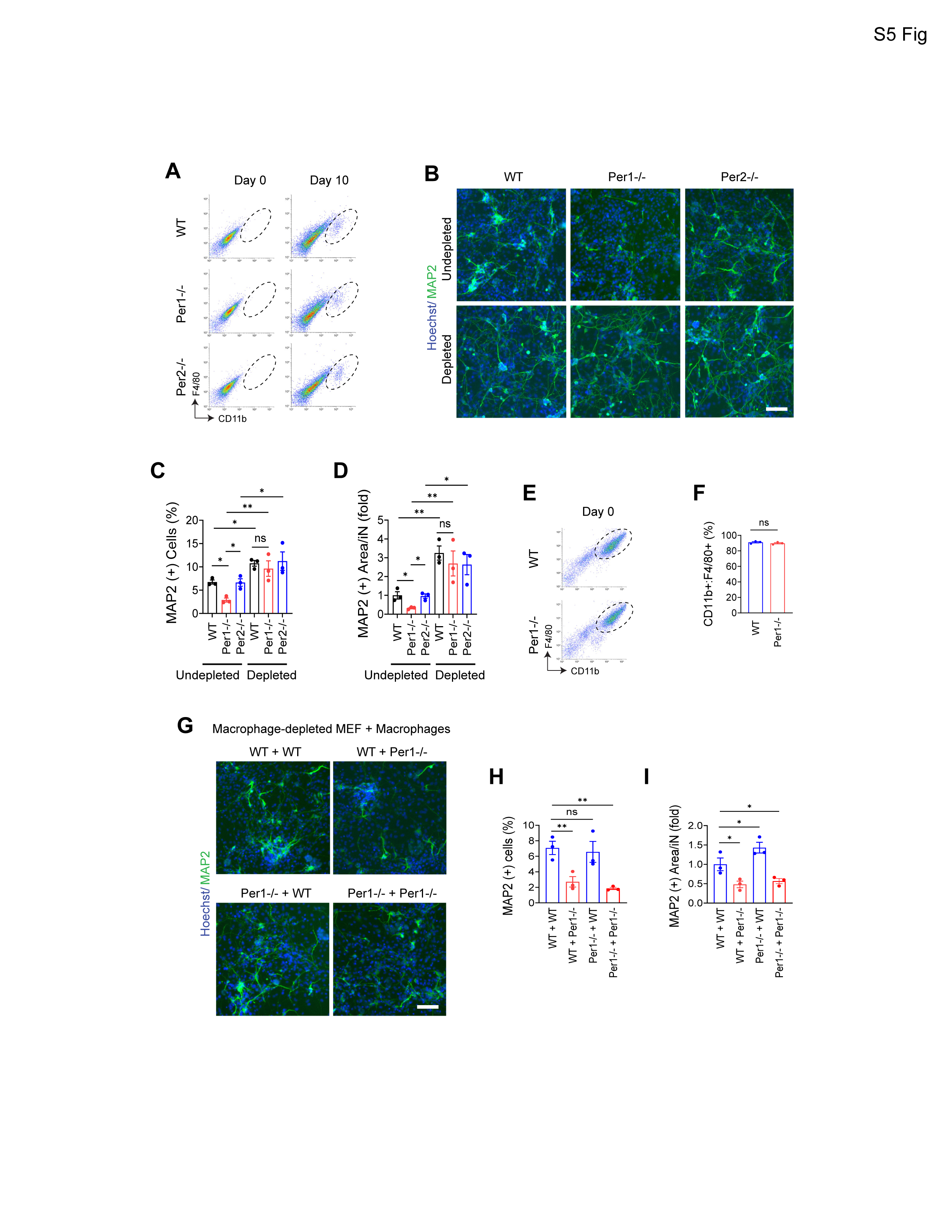

Supplement: S5 Fig — (A) Flow cytometry to quantify the macrophage fractions in macrophage-depleted MEFs during iN reprogramming. (B) Immunofluorescence staining of iNs with MAP2 antibody on day 11. (C) Percentages of MAP2 (+) cells related to (B). (D) MAP2 (+) neurite area in each MAP2 (+) cell related to (B). The value with undepleted WT cells was defined as 1.0. (E) Flow cytometry to quantify purified macrophage fractions. (F) Percentages of purified macrophages on day 0. (G) Immunofluorescence staining of iNs with MAP2 antibody on day 11 after mixing macrophage-depleted MEFs (before “+”) and macrophages (after “+”) of 2 genotypes each. (H) Percentages of MAP2 (+) cells related to (G). (I) MAP2 (+) neurite area in each MAP2 (+) cell related to (G). * p < 0.05 and ** p < 0.01 with ordinary one-way ANOVA with Bonferroni’s multiple comparison test. (F) Used two-tailed t test; ns indicates statistically not significant. All data are based on biological triplicates with technical triplicates each. The data underlying this figure can be found in S1 Data. (TIF) [file pbio.3002419.s005.tif]

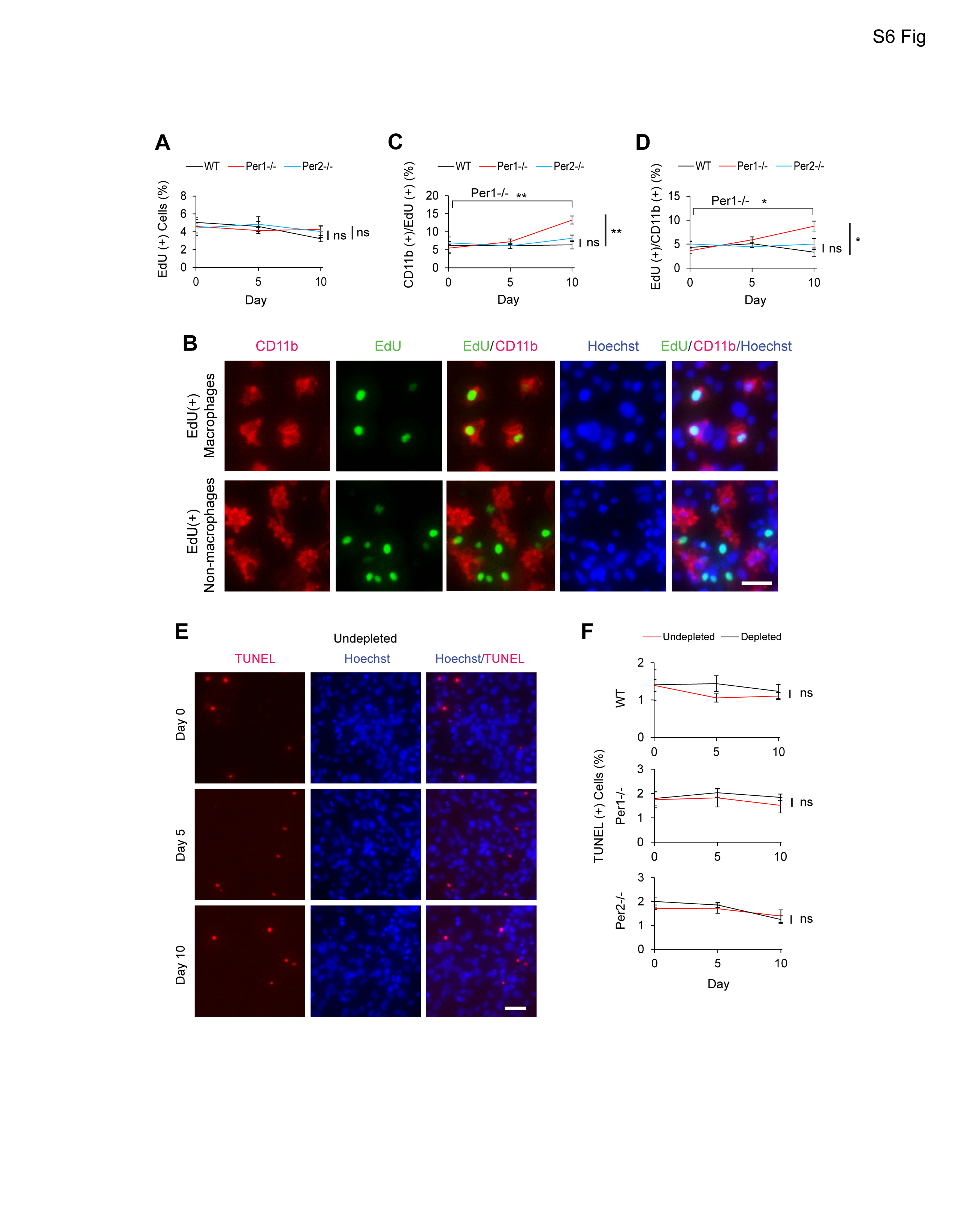

Supplement: S6 Fig — (A) The frequency of EdU (+) cells during iN reprogramming. (B) Representative images of EdU uptake into macrophages (top) and non-macrophages (bottom). (C) The frequency of macrophages within the EdU (+) population during the reprogramming. (D) The frequency of EdU (+) cells within the macrophage population during the reprogramming. (E) TUNEL staining of undepleted WT cells during the reprogramming. (F) The frequency of TUNEL (+) cells in undepleted and macrophage-depleted cells during the reprogramming. There was no statistically significant difference between each genotype on day 10. Bar, 50 μm. * p < 0.05 and ** p < 0.01 with ordinary one-way ANOVA with Bonferroni’s multiple comparison test. (F) Used two-tailed t test; ns indicates statistically not significant. All data were based on biological triplicates with technical triplicates each. The data underlying this figure can be found in S1 Data. (TIF) [file pbio.3002419.s006.tif]

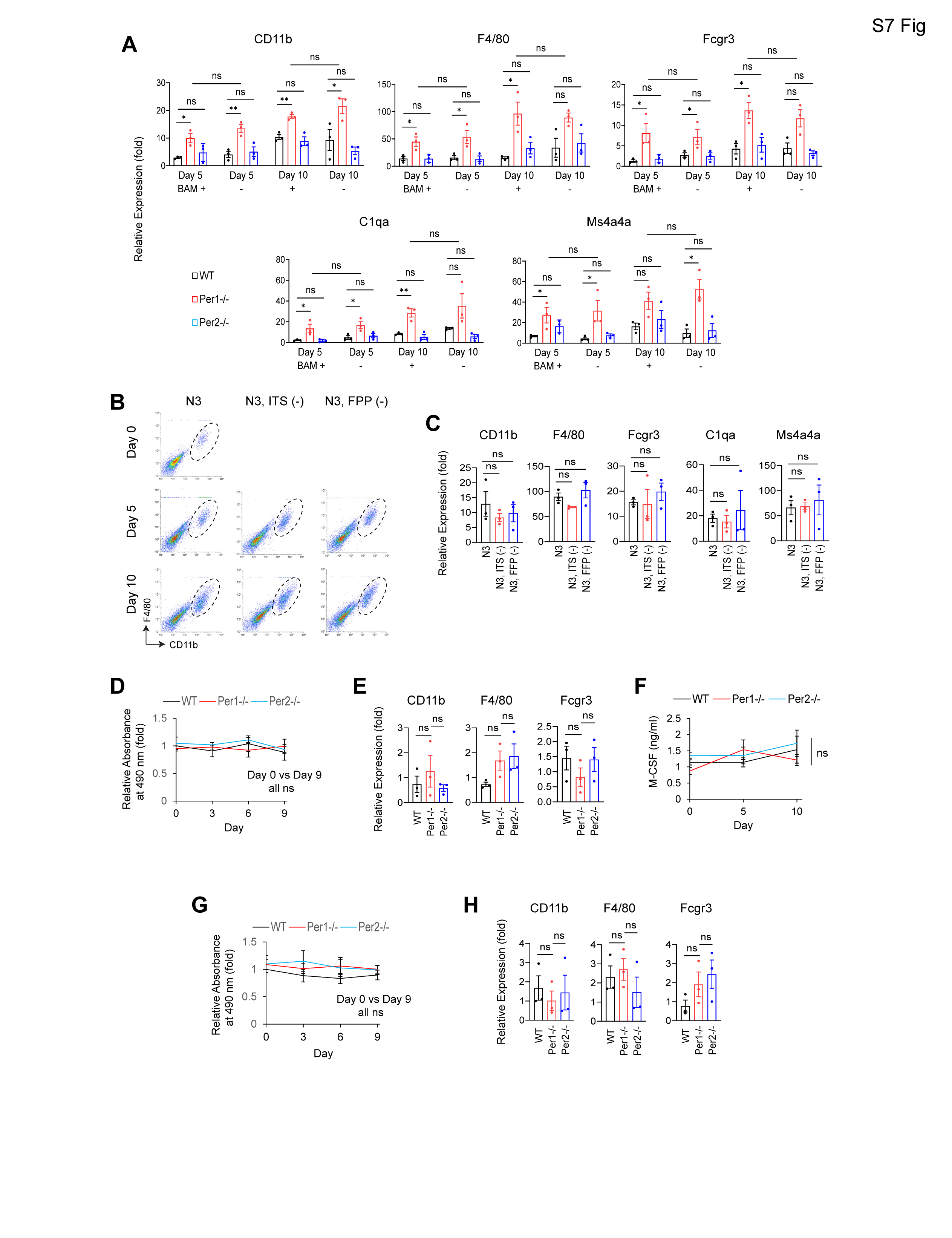

Supplement: S7 Fig — (A) PCR of macrophage genes upon culture of MEFs with and without BAM viruses. The values obtained with WT MEFs without virus on day 0 were defined as 1.0. (B) Flow cytometry of Per1-/- MEFs after culture in N3, N3 without ITS (N3, ITS (-)), and N3 without FGF2, progesterone, and putrescine (N3, FPP (-)) in the absence of viruses. (C) PCR of macrophages genes on day 10 after culture of MEFs under the conditions in (B). (D) MTS assays following the culture of purified macrophages in the N3 medium without BAM viruses. The values with WT cells on day 0 was defined as 1.0. (E) PCR of macrophage marker genes after culture of purified macrophages in the N3 medium without BAM viruses for 9 days. The values obtained with WT macrophages on day 0 were defined as 1.0. (F) ELISA of M-CSF in the supernatant during iN reprogramming. The culture medium was not replaced for 2 days before harvest. (G) MTS assays following the culture of purified macrophages in the N3 medium with 2 ng/ml M-CSF. The values with WT cells on day 0 was defined as 1.0. (H) PCR of macrophage marker genes after culture of purified macrophages in the N3 medium with 2 ng/ml M-CSF for 9 days. The values obtained with WT macrophages on day 0 were defined as 1.0. * p < 0.05 and ** p < 0.01 with ordinary one-way ANOVA with Bonferroni’s multiple comparison test; ns indicates statistically not significant. All data were based on biological triplicates with technical triplicates each. The data underlying this figure can be found in S1 Data. (TIF) [file pbio.3002419.s007.tif]

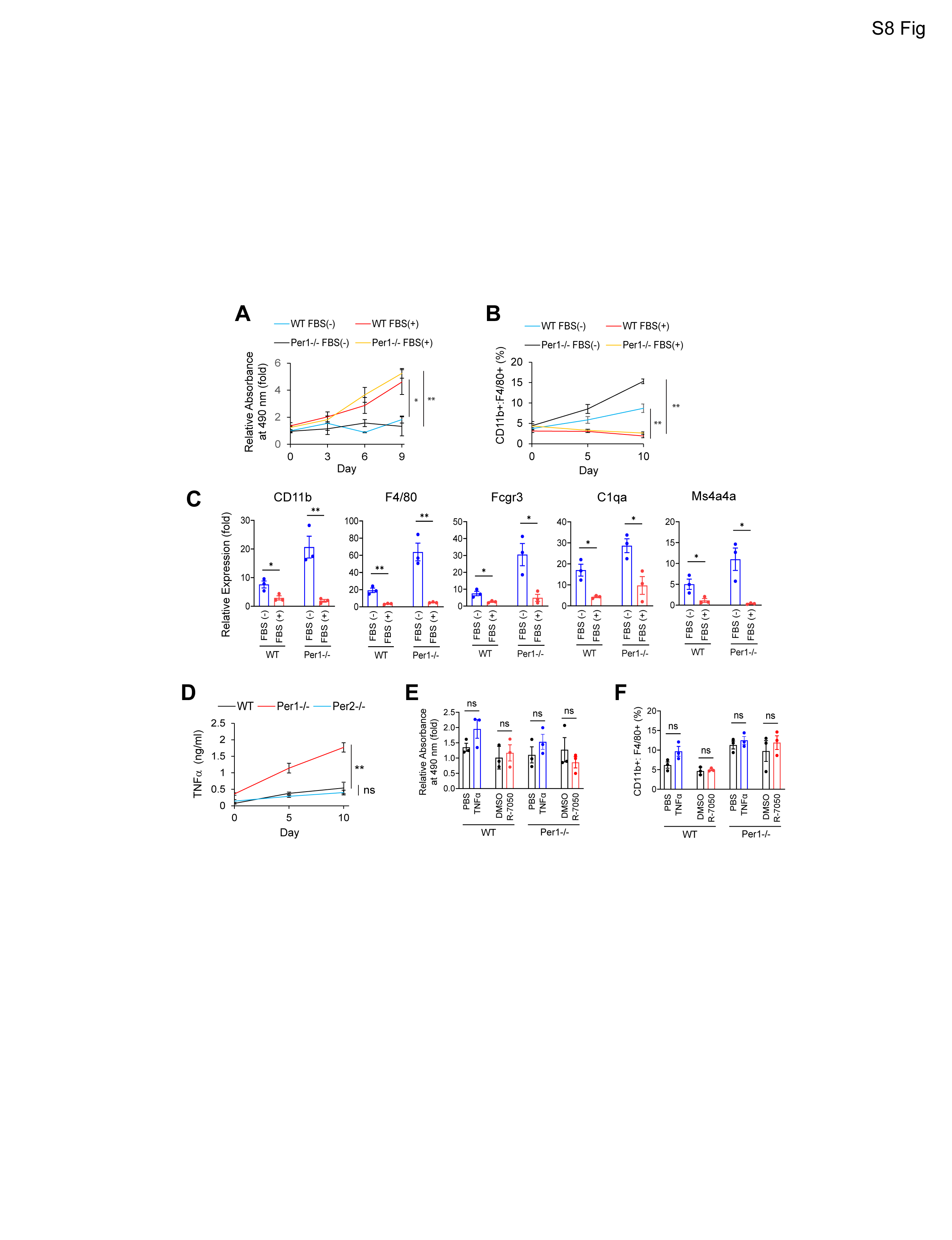

Supplement: S8 Fig — (A) MTS assays following the culture of WT and Per1-/- MEFs with and without FBS in the absence of viruses. The values with WT MEFs without FBS was defined as 1.0. (B) Macrophage fractions related to (A). (C) PCR of macrophage genes on day 10 related to (B). (D) ELISA of TNFα in the supernatants during iN reprogramming. (E) MTS assays studying the effects of TNFα and R-7050 on day 10. The values on day 0 were defined as 1.0. (F) The effects of TNFα and R-7050 on the macrophage fractions on day 10. PBS and DMSO were used as controls for TNFα and R-7050, respectively. * p < 0.05 and ** p < 0.01 with ordinary one-way ANOVA with Bonferroni’s multiple comparison test (A, B, and D) or with two-tailed t test (C, E, and F); ns indicates statistically not significant. All data were based on biological triplicates with technical triplicates each. The data underlying this figure can be found in S1 Data. (TIF) [file pbio.3002419.s008.tif]
