# Supplementary material for: The circadian regulator PER1 promotes cell reprogramming by inhibiting inflammatory signaling from macrophages
Source: PLoS Biol. 2023 Dec 4;21(12):e3002419. doi: 10.1371/journal.pbio.3002419 (PMC10721173; doi:10.1371/journal.pbio.3002419)
Supplement: S2 Table — (PDF) [file pbio.3002419.s010.pdf]

S2 Table. Sequences of PCR primers

Gene	Forward	Reverse
Gapdh	TGCACCACCAACTGCTTAG	GATGCAGGGATGATGTTC
CD11b	GCTTCAGAGATGACCAGTAAGG	ACAGGGATCCAGAAGACTACA
F4/80	CGTCAGGTACGGGATGAATATAAG	ATCTTGGAAGTGGATGGCATAG
Fcgr3	CAACCCTGGGAACTCTTCTAC	GTCATTGACTGTGGCCTTAAAC
C1qa	GTGCCCGGCTTCTATTACTT	CCCGGAGGAAGACTTGATAAAC
Ms4a4a	CTGCATAGGAGTATCCCTCTCT	GTCTCTGCCTTGGTAGGATTTG
Tnf	TTGTCTACTCCCAGGTTCTCT	GAGGTTGACTTTCTCCTGGTATG
Tlr2	CACTATCCGGAGGTTGCATATC	GGAAGACCTTGCTGTTCTCTAC
Ccl2	CTCACCTGCTGCTACTCATTC	ACTACAGCTTCTTTGGGACAC
Il6	CTTCCATCCAGTTGCCTTCT	CTCCGACTTGTGAAGTGGTATAG
Nos2	GGAATCTTGGAGCGAGTTGT	CCTCTTGTCTTTGACCCAGTAG
Il1b	CCACCTCAATGGACAGAATATCA	CCCAAGGCCACAGGTATTT
Retnla	GCTGATGGTCCCAGTGAATA	CGTTACAGTGGAGGGATAGTTAG
Chil3	GCTAAGGACAGGCCAATAGAA	GCATTCCAGCAAAGGCATAG
Arg1	GTCCCTAATGACAGCTCCTTTC	CCACACTGACTCTTCCATTCTT
CD163	CAGACTGGTTGGAGGAGAAATC	CAGCTTCCAGAGACAAGTCAA
Clec4e	GGTTATCGACACACAGGAAGAG	GGGACTCTGTGAAAGGTGTATC
Cxcl13	CGGTATTCTGGAAGCCCATTA	GGCGTAACTTGAATCCGATCTA
Marco	TGGGAAGTAAAGAACTCCTCAAA	GTCTCCATCACAGGGAACATAG
