# Supplementary material for: The circadian regulator PER1 promotes cell reprogramming by inhibiting inflammatory signaling from macrophages
Source: PLoS Biol. 2023 Dec 4;21(12):e3002419. doi: 10.1371/journal.pbio.3002419 (PMC10721173; doi:10.1371/journal.pbio.3002419)

Original images of western blotting - 1

Whereas X-ray films were used in Fig 5C and S1A Fig, the iBright imaging system (Thermo Fisher Scientific) was used in Fig 5F, wihch made the background more white than that in X-ray film images, giving an artificial impression. To address this issue, overlaid images of bright field and chemiluminescence images are also shown to display membrane contours in Fig 5F.

Fig 5C

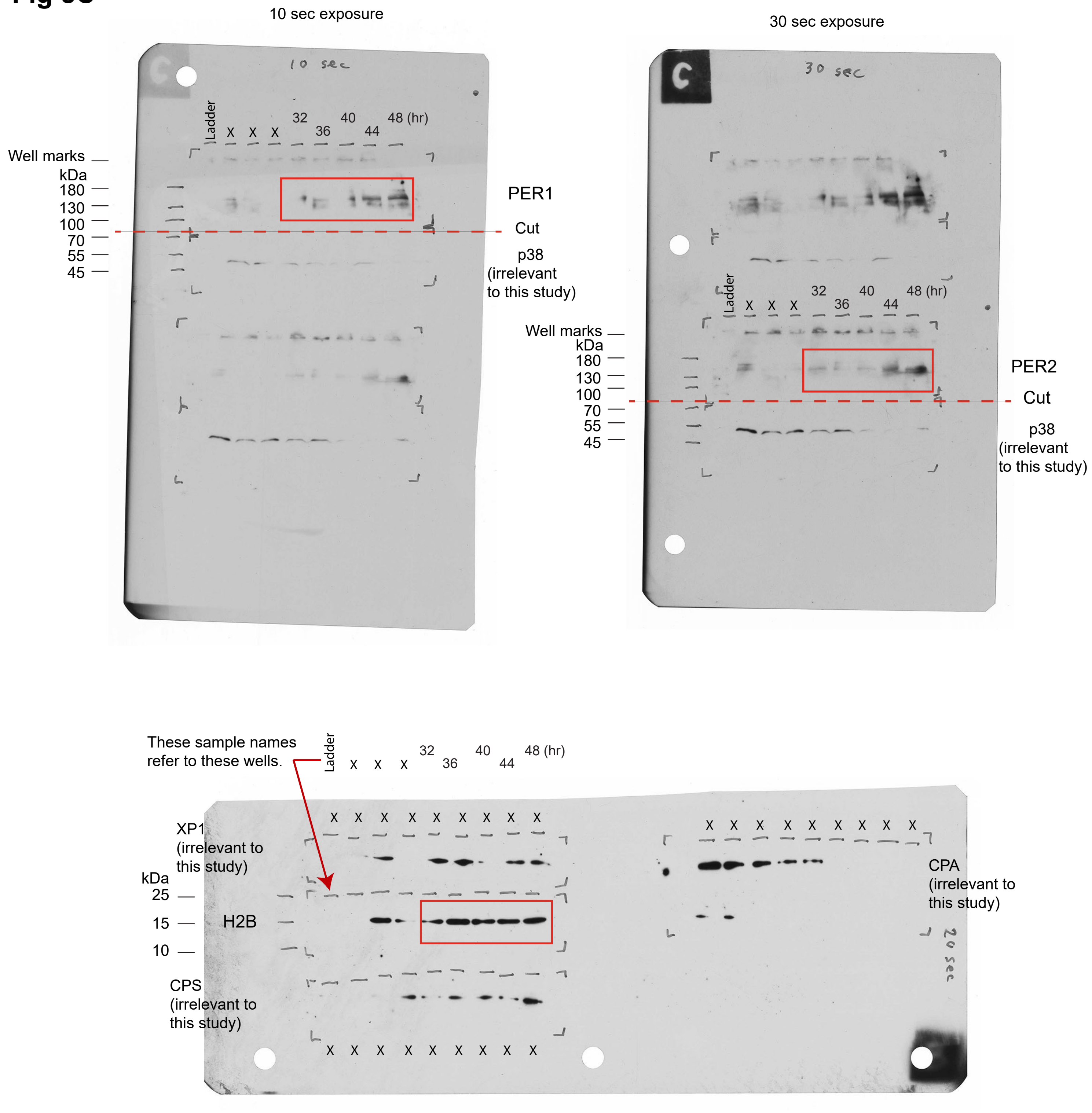

Original images of western blotting - 2

Fig 5F

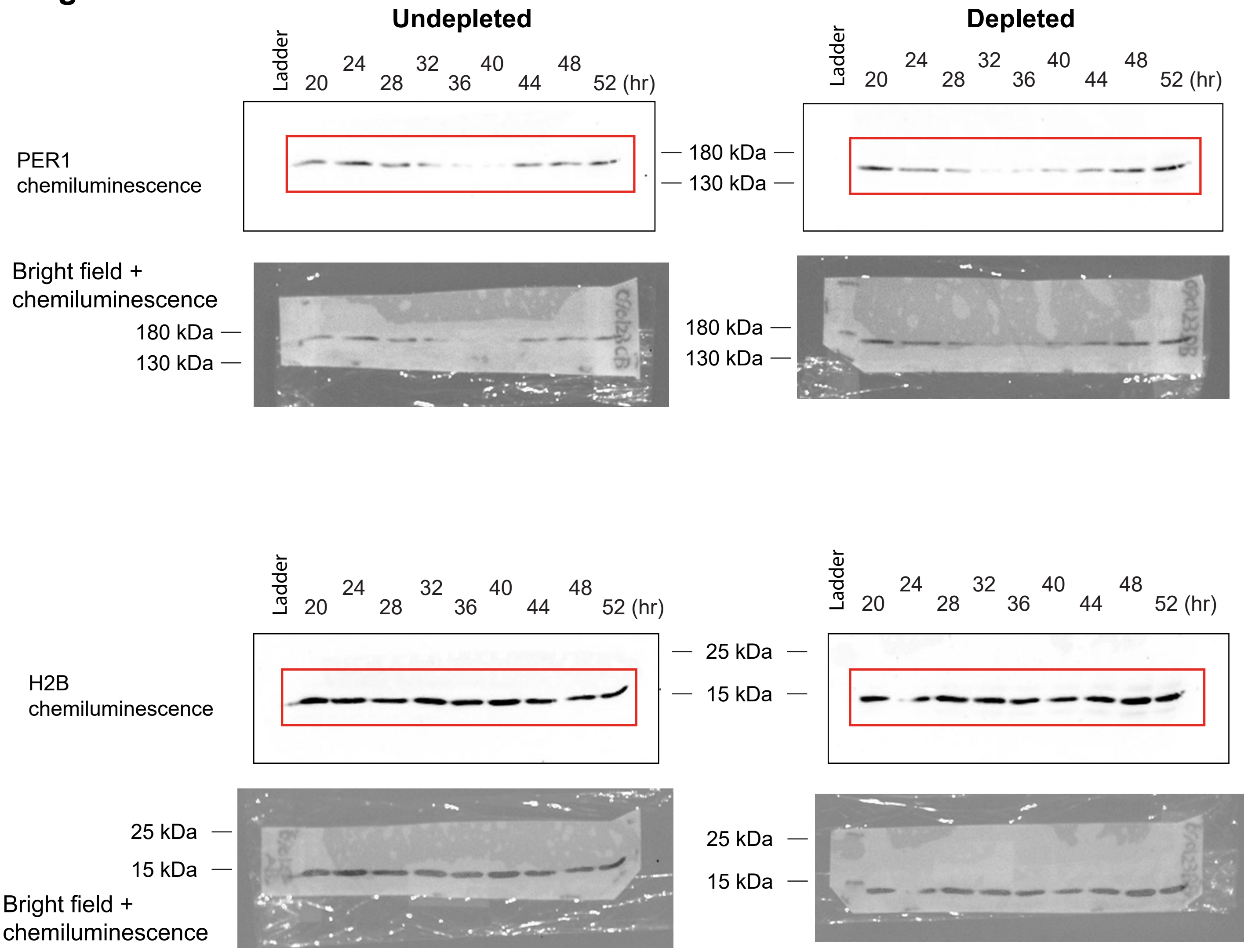

Original images of western blotting - 3

The membranes were cut into three pieces each and the top and the bottom were separately stained.

S1A Fig

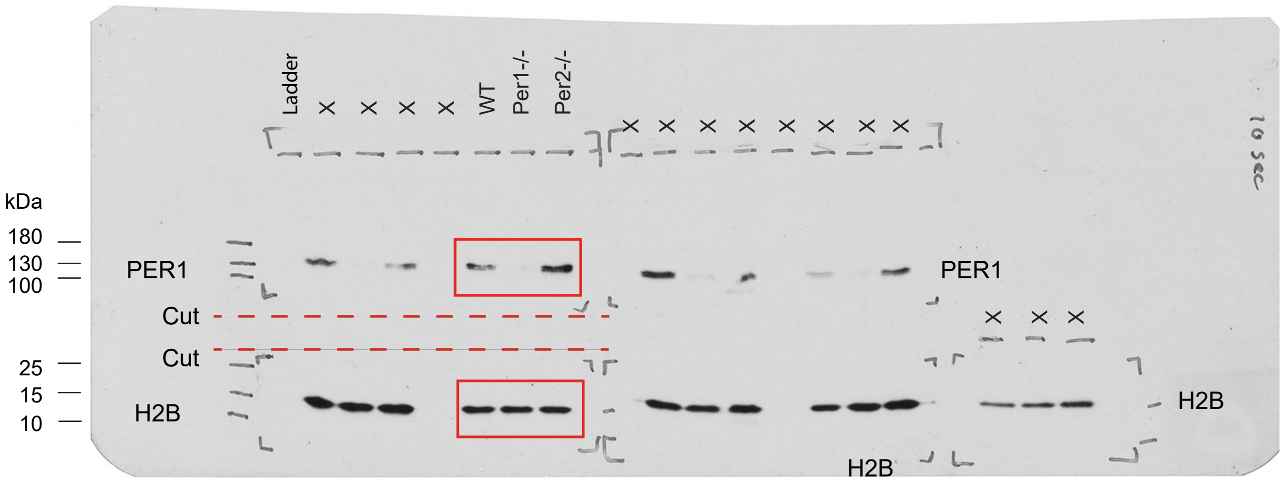

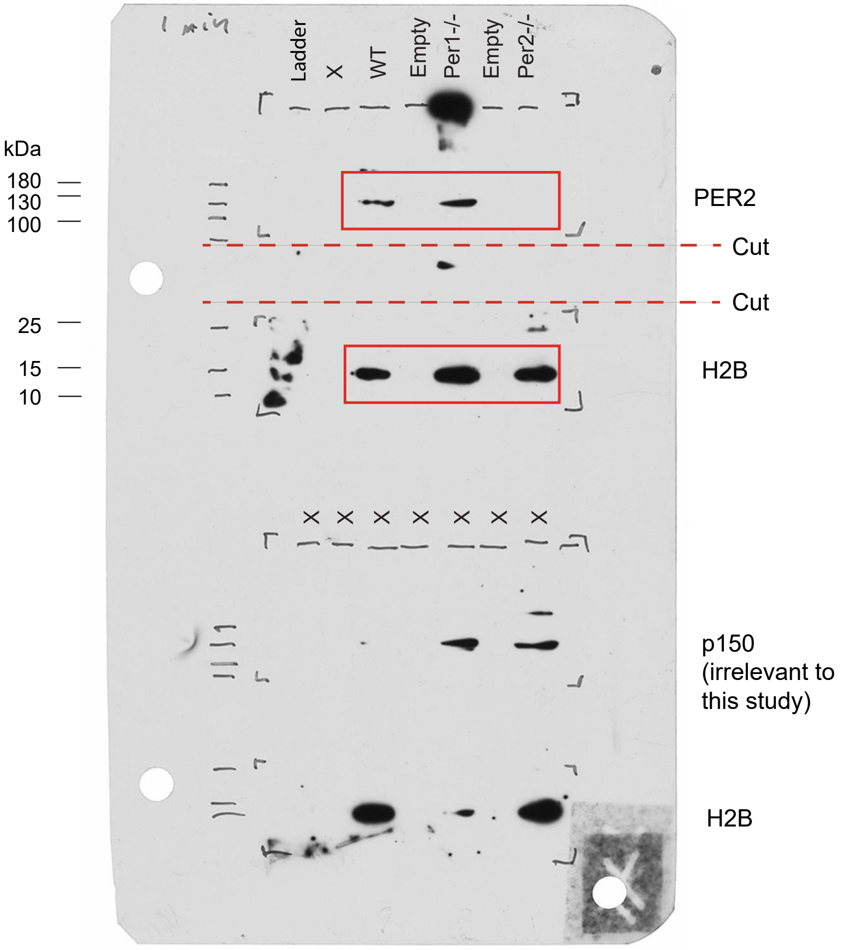

Supplement: S1 Raw Images — (PDF) [file pbio.3002419.s012.pdf]
